# Supplementary material for: Crystal orientation fabric anisotropy causes directional hardening of the Northeast Greenland Ice Stream
Source: Nat Commun. 2023 May 8;14:2653. doi: 10.1038/s41467-023-38139-8 (PMC10167229; doi:10.1038/s41467-023-38139-8)
Supplement: Supplementary file 1 — Supplementary Information [file 41467_2023_38139_MOESM1_ESM.pdf]

Crystal orientation fabric anisotropy causes directional hardening of the Northeast Greenland Ice Stream

Tamara Annina Gerber[1*], David Armond Lilien[2], Nicholas Mossor Rathmann[1], Steven Franke[3], Tun Jan Young[4,5], Fernando Valero-Delgado[3], M. Reza Ershadi[6], Reinhard Drews[6], Ole Zeising[3], Angelika Humbert[3,7], Nicolas Stoll[3,7], Ilka Weikusat[3,6], Aslak Grinsted[1], Christine Schøtt Hvidberg[1], Daniela Jansen[3], Heinrich Miller[3], Veit Helm[3], Daniel Steinhage[3], Charles O'Neill[8], John Paden[9], Siva Prasad Gogineni[10], Dorthe Dahl-Jensen[1,2], and Olaf Eisen[3,7*]

[*]**corresponding authors:** tamara.gerber@nbi.ku.dk, olaf.eisen@awi.de

[1]*Section for the Physics of Ice, Climate and Earth, Niels Bohr Institute, University of Copenhagen, Copenhagen, Denmark*

[2]*Centre for Earth Observation Science, University of Manitoba, Winnipeg, Canada*

[3]*Alfred Wegener Institute, Helmholtz Centre for Polar and Marine Research, Bremerhaven, Germany*

[4]*Scott Polar Research Institute, University of Cambridge, Cambridge, United Kingdom*

[5]*School of Geography & Sustainable Development, University of St Andrews, St Andrews KY16 9AL, United Kingdom.*

[6]*Department of Geosciences, Tübingen University, Tübingen, Germany*

[7]*Department of Geosciences, University of Bremen, Bremen, Germany*

[8]*EH Group Inc., Tuscaloosa, USA*

[9]*Centre for Remote Sensing of Ice Sheets (CReSIS), University of Kansas, Lawrence, USA*

[10]*Remote Sensing Centre, University of Alabama, Tuscaloosa, USA*

1 Analytical methods

The propagation of radar waves is determined by the complex relative dielectric constant

$$\varepsilon^* = \varepsilon' - i\frac{\gamma}{\omega\varepsilon_0}, \tag{1}$$

where the real part, ε', is the relative dielectric permittivity, and the imaginary part corresponds to the dielectric loss factor depending on the electrical conductivity, γ, the angular frequency, ω, and the dielectric permittivity of free space, ε_0. For simplicity, primes are omitted henceforth, and all ε symbols refer to the relative dielectric permittivity unless stated otherwise. The conductivity of ice mainly depends on its impurity content and temperatures, so the imaginary part of the dielectric permittivity constant can be assumed to be isotropic for near–vertical incidence. In contrast, the bulk permittivity shows directional dependence related to the COF.

Ice crystals show uniaxial birefringence, so the real part of the relative dielectric permittivity tensor of a monocrystal (superscript m) can be written as

$$\boldsymbol{\varepsilon}^{\mathrm{m}} = \begin{pmatrix} \varepsilon_\perp^{\mathrm{m}} & 0 & 0 \\ 0 & \varepsilon_\perp^{\mathrm{m}} & 0 \\ 0 & 0 & \varepsilon_\parallel^{\mathrm{m}} \end{pmatrix}, \tag{2}$$

where $\varepsilon_\perp^{\mathrm{m}}$ and $\varepsilon_\parallel^{\mathrm{m}}$ are the relative dielectric permittivities perpendicular and parallel to the crystal c-axis. At typical radar frequencies in the range of 1 MHz to 1 GHz, the dielectric anisotropy of a monocrystal, $\Delta\varepsilon^{\mathrm{m}} = \varepsilon_\parallel^{\mathrm{m}} - \varepsilon_\perp^{\mathrm{m}}$, is approximately $\Delta\varepsilon^{\mathrm{m}} \simeq 0.034 - 0.035$ [1].

In polycrystalline ice, the relative permittivity of the bulk tensor (no superscript) relates to the single ice crystal as follows (e.g. Fujita *et al.* [2]):

$$\boldsymbol{\varepsilon} = \begin{pmatrix} \varepsilon_{\mathrm{x}} & 0 & 0 \\ 0 & \varepsilon_{\mathrm{y}} & 0 \\ 0 & 0 & \varepsilon_{\mathrm{z}} \end{pmatrix} = \begin{pmatrix} \varepsilon_\perp^{\mathrm{m}} + \Delta\varepsilon^{\mathrm{m}}\lambda_{\mathrm{x}} & 0 & 0 \\ 0 & \varepsilon_\perp^{\mathrm{m}} + \Delta\varepsilon^{\mathrm{m}}\lambda_{\mathrm{y}} & 0 \\ 0 & 0 & \varepsilon_\perp^{\mathrm{m}} + \Delta\varepsilon^{\mathrm{m}}\lambda_{\mathrm{z}} \end{pmatrix}, \tag{3}$$

where ε_{x}, ε_{y} and ε_{z} correspond to the directional permittivities. Here, the coordinate system represents the principal COF axes, whose lengths are determined by the COF eigenvalues λ_{x}, λ_{y} and λ_{z}.

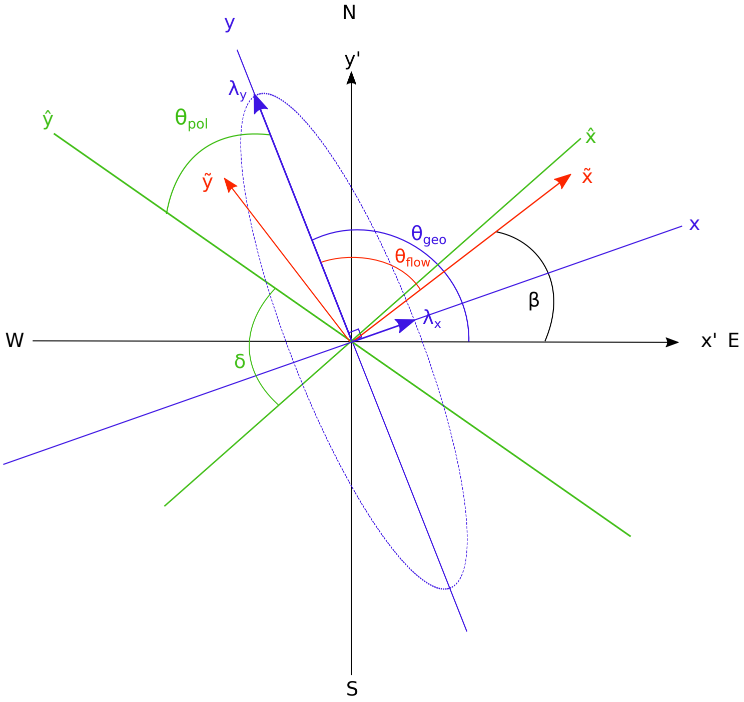

Figure 1: **Coordinate systems used in this study.** Geographical coordinates (black) are denoted as x′ and y′, equalling polar stereographic East and North respectively. Ice flows in x̃–direction (red), defined from geographic east counterclockwise by angle β. θ_{flow} is the angle between the flow direction and the eigenvector corresponding to the larger horizontal eigenvalue of the crystal orientation fabric. The Eigenframe (blue) is denoted by x and y, whereby x points in the direction of the smaller horizontal eigenvalue. Radar waves at crosspoints are polarised in x̂ and ŷ respectively (green) and related to the Eigenframe by angle θ_{pol}. δ is the smaller angle between the polarisation directions. θ_{geo} is the angle between geographic East and the larger horizontal eigenvalue.

The orientation of the COF in ice sheets is related to deformation through ice flow, but the ability to determine its strength and type strongly depends on its orientation relative to the radar wave polarisation. To describe these relations, we introduce four coordinate systems (Fig. 1) where x′ and y′ are the geographic coordinates, x̃ and ỹ point parallel and perpendicular to the ice flow direction, x̂ and ŷ describe the polarisation directions of two orthogonal radar waves, and x and y denote the COF Eigenframe, as mentioned above. For nadir surveys, the radar waves transmitted at the surface of ice sheets travel vertically through the ice column and are polarised perpendicular to the direction of propagation. The radar wave speed is essentially determined by the bulk dielectric permittivity in the polarisation direction, hence, radio-echo sounding (RES) is only sensitive toward horizontal variations of the relative dielectric permittivity:

$$\Delta\varepsilon = \varepsilon_{\text{y}} - \varepsilon_{\text{x}} = \Delta\varepsilon^{\text{m}}(\lambda_{\text{y}} - \lambda_{\text{x}}) = \Delta\varepsilon^{\text{m}}\Delta\lambda. \tag{4}$$

We, therefore, assume the vertical direction, z, is consistent for all coordinate systems and define it as depth below the ice surface.

The electromagnetic wave speed, c, is determined by the complex dielectric constant as follows:

$$c = \frac{c_0}{\sqrt{\varepsilon *}} \approx \frac{c_0}{\sqrt{\varepsilon}}, \tag{5}$$

where c_0 is the speed of light in free space. Since the real part of the relative dielectric constant of ice is several orders of magnitude larger than the dielectric loss factor, the latter has a diminishing effect, so the wave speed primarily depends on ε. Radar waves are decomposed into ordinary and extraordinary wave components when travelling through polycrystalline ice with bulk anisotropic dielectric properties. If the dielectric permittivity varies between the two polarisation directions, i.e. $\varepsilon_{\hat{x}} \neq \varepsilon_{\hat{y}}$, the wave speeds and wavelengths of two radar waves polarised in x̂ and ŷ differ

slightly. Consequently, the travel time difference, Δt, between the reflected waves with different antenna polarisations is related to the horizontal anisotropy of the ice. In the following sections, we describe the individual methods we used to derive horizontal anisotropy from signatures caused by radar wave decomposition.

1.1 Travel-time analysis of radar crosspoints

The EGRIP-NOR-2018 radar data set [3] was recorded as a dense grid across the ice stream with antennas polarised parallel to the flight direction (HH), so at each radar line crosspoint, two radar traces with near-orthogonal antenna polarisations lie close to each other. The travel-time difference of a reflection, Δt, between two orthogonally polarised waves is:

$$\Delta t = t_{\hat{y}} - t_{\hat{x}} = \frac{2(\sqrt{\varepsilon_{\hat{y}}} - \sqrt{\varepsilon_{\hat{x}}})}{c_0} z, \tag{6}$$

where $t_{\hat{x}}$ and $t_{\hat{y}}$ are the recorded two-way travel times in the corresponding polarisation directions, z is the reflector depth, $\varepsilon_{\hat{y}}$ and $\varepsilon_{\hat{x}}$ are the directional relative permittivities of the ice above the reflecting interface and the factor of 2 results from the two-way travel path.

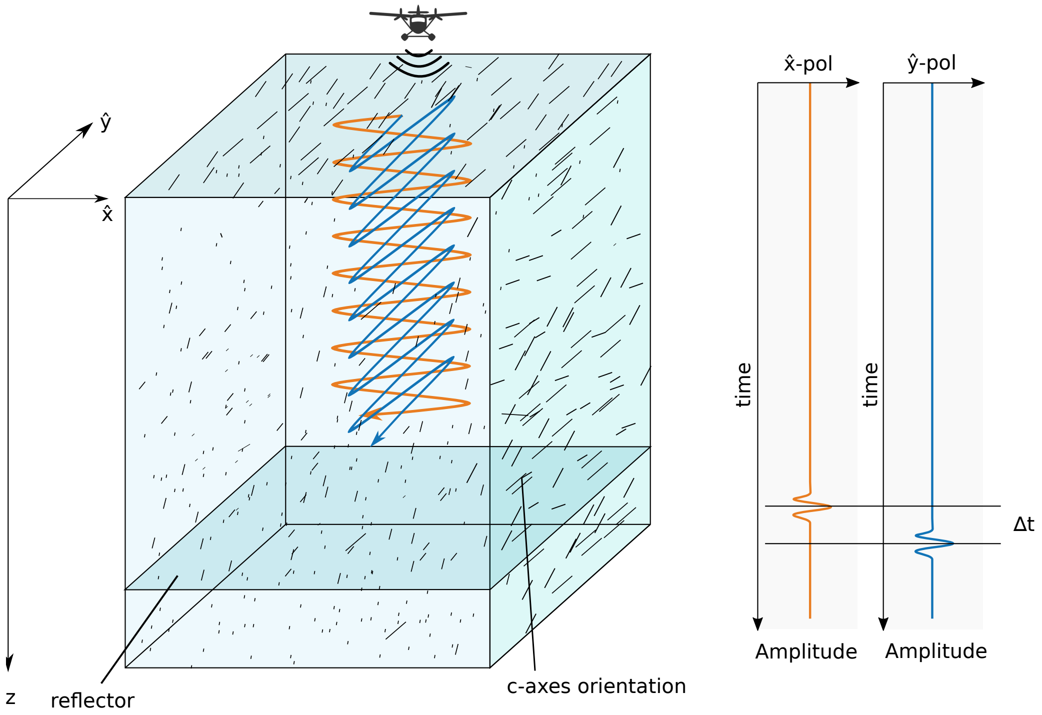

Figure 2: **Illustration of travel-time difference in a horizontally anisotropic media.** A reflecting interface at depth z and a horizontally anisotropic crystal orientation fabric (COF) leads to a travel time difference (Δt) of the reflected signal for different wave polarisations $\hat{x}$ and $\hat{y}$. In this example, the c-axes are predominantly oriented in the $\hat{y}$-direction, so the $\hat{y}$-polarised wave travels at a slightly slower velocity and the reflection signal is delayed in comparison to the $\hat{x}$-polarised signal.

Travel–time picking and time-depth conversion

We manually picked $t_{\hat{x}}$ and $t_{\hat{y}}$ of prominent reflections observed in the closest traces of crossing radar profiles with intersection angles of approximately 90°. To account for eventual differences in the flight height of the aircraft, we shifted time zero to the first break of the air–ice interface. We moreover used a second-order spline interpolation to increase the temporal resolution from 0.033 µs to 0.001 µs.

In the EGRIP ice core, the relative dielectric permittivity was inferred from Dielectric Profiling (DEP) by Mojtabavi *et al.* [4] and was used here to estimate the electromagnetic wave-speed profile

through the ice column. In the firn layers, the dielectric permittivity is controlled by the density and increases with depth. Below the transition into pure ice, changes in the permittivity, and thus also in the electromagnetic wave speed, occur mainly due to the COF. However, the instrument sensitivity of the DEP device is not high enough to measure these effects and permittivity variations below the transition into pure ice are related to instrument noise [5]. To estimate the radar wave speeds, we assumed a firn permittivity of 1.55 [6] at the ice-sheet surface and extrapolated the smoothed DEP profile (moving average with 5 m window length) from the core onset (13 m depth) to the surface. Below the transition into pure ice at ∼200 m we assumed a constant relative permittivity of 3.15. Although the DEP-inferred wave-speed profile is a reasonable representation at EGRIP, we recognise that variations and inhomogeneities in firn densification can lead to spatially varying wave speed profiles. While temperature and accumulation rate only vary mildly over distances relevant in this study, increased deformation accelerates densification in the shear margins (e.g. Riverman *et al.* [7], Vallelonga *et al.* [8], Christianson *et al.* [9], and Oraschewski & Grinsted [10]). Consequently, the vertical density and electromagnetic wave speed gradients can be expected to be stronger in the vicinity of the shear margins. Assuming a spatially constant wave speed profile could therefore affect the calculation of reflector depths at crosspoints in the immediate vicinity of the shear margins. The firn correction amounts to some 10 m, which represents the most conservative estimate possible (i.e. completely neglecting the influence of higher wave speeds in firn). Most of the crosspoints analysed here are more than 2 km away from the shear zone, though, so the spatially uniform wave speed assumption is arguably justified in most cases.

The depth of the picked reflections was estimated by converting the average travel times $\bar{t} = \frac{1}{2}(t_{\hat{x}} + t_{\hat{y}})$ to depth, using the DEP-based wave-speed profile. From Equation (6) we defined a maximum threshold for the depth-dependent travel-time difference resulting from a horizontal single maximum COF, assuming that $\varepsilon^{\mathrm{m}}_{\parallel} = 3.167$ and $\varepsilon^{\mathrm{m}}_{\perp} = 3.133$, i.e. $\Delta\varepsilon^{\mathrm{m}} = 0.034$ [1]. Picked reflections where the corresponding threshold of Δt is exceeded were considered outliers and discarded. We further removed picked reflections that deviate by more than two standard deviations from the trend of the time–depth profile. Crosspoints, where less than five reflections could be clearly identified, were excluded from further analyses.

Deriving the apparent horizontal crystal orientation fabric anisotropy

In a layered media, the picked two-way travel times $(t_{\hat{x}}, t_{\hat{y}})$ of two waves reflected at layer i and polarised in $\hat{x}$ and $\hat{y}$ directions respectively, are given by:

$$t_{\hat{x}} = \frac{2}{c_0}\sum_{i=1}^{I}\sqrt{\varepsilon_{\hat{x},i}}(z_i - z_{i-1}) \text{ and } t_{\hat{y}} = \frac{2}{c_0}\sum_{i=1}^{I}\sqrt{\varepsilon_{\hat{y},i}}(z_i - z_{i-1}). \tag{7}$$

While inferring the depth-varying permittivities from Equation (7) is possible in theory, it is unfeasible here due to the relatively large uncertainties on $t_{\hat{x}}$ and $t_{\hat{y}}$ (elaborated on below). By replacing $\varepsilon_{\hat{x}}$ and $\varepsilon_{\hat{y}}$ with the depth-averaged permittivities $\overline{\varepsilon_{\hat{x}}}$ and $\overline{\varepsilon_{\hat{y}}}$, Equation (7) can be linearised:

$$t_{\hat{x}} = \frac{2\sqrt{\overline{\varepsilon_{\hat{x}}}}}{c_0}z = m_{\hat{x}}z \text{ and } t_{\hat{y}} = \frac{2\sqrt{\overline{\varepsilon_{\hat{y}}}}}{c_0}z = m_{\hat{y}}z. \tag{8}$$

By determining the slopes $m_{\hat{x}}$ and $m_{\hat{y}}$ from a linear regression through the picked travel times, the depth-averaged apparent horizontal dielectric anisotropy ($\Delta\varepsilon_{\mathrm{a}}$) can be derived:

$$\Delta\varepsilon_{\mathrm{a}} = \overline{\varepsilon_{\hat{x}}} - \overline{\varepsilon_{\hat{y}}} = \frac{c_0^2}{4}(m_{\hat{x}}^2 - m_{\hat{y}}^2), \tag{9}$$

which relates to the apparent difference in the horizontal eigenvalues:

$$\Delta\lambda_{\mathrm{a}} = \lambda_{\hat{x}} - \lambda_{\hat{y}} = \frac{\Delta\varepsilon_{\mathrm{a}}}{\Delta\varepsilon^{\mathrm{m}}}. \tag{10}$$

We emphasise that the apparent horizontal anisotropy ($\Delta\lambda_{\mathrm{a}}$) inferred by this method is not equivalent to the absolute horizontal COF anisotropy ($\Delta\lambda$) if the polarisation of the two radar traces does

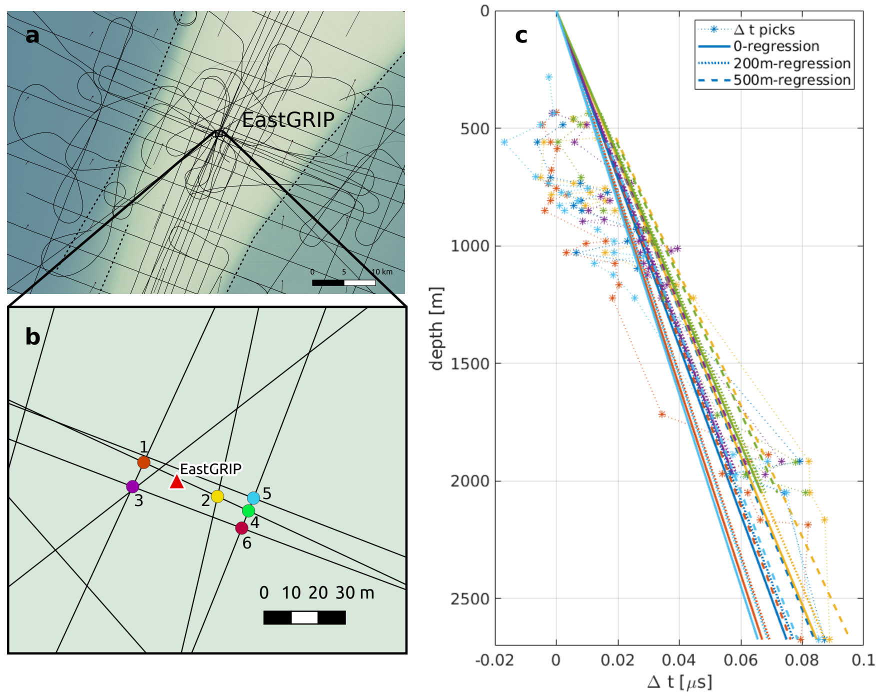

Figure 3: **Uncertainty analysis of travel-time difference method.** **a** Overview and **b** location of six radar crosspoints near the East Greenland ice-core project (EastGRIP). **c** Picked travel time differences between internal reflections in along-flow and across-flow polarised radargrams versus depth (asterisk symbols) and the fit of a first-degree polynomial through the origin (solid line), 200 m (dotted line) and 500 m (dashed line) from which the apparent horizontal anisotropies $\Delta\lambda_a^0$, $\Delta\lambda_a^{200}$ and $\Delta\lambda_a^{500}$ in Table 1 were derived.

not align with the two horizontal eigenvectors. $\Delta\lambda_a$ can thus be regarded as a lower-bound estimate of the horizontal anisotropy.

To evaluate the robustness of our method, we compared six crosspoints close to the EGRIP drill site that are separated by less than 50 m from each other. Within this distance, COF variations can be assumed to be negligible. We investigated the sensitivity to varying depths by employing three linear regressions through different depth points at 0 m, 200 m and 500 m. The corresponding travel times at 200 m and 500 m were calculated from the $\overline{\varepsilon_{\hat{x}}}$ and $\overline{\varepsilon_{\hat{y}}}$ inferred from the regression through the origin. The regressions running through 200 m and 500 m result in higher horizontal anisotropy values due to the higher relative weight of greater depths, where the COF is likely to be more pronounced (Table 1). Table 2 shows an example of this analysis for crosspoint 3.

Uncertainties in travel-time analysis

Most uncertainties in this method arise from determining the travel times $t_{\hat{x}}$ and $t_{\hat{y}}$. The range resolution of the recorded signal corresponds to a temporal resolution of 0.033 µs, but by interpolating the waveform within the sampling interval, we reduced the picking uncertainty to roughly ± 0.01 µs. The radar traces were not recorded concurrently in space and time, so differences in background noise can arise from variations in weather conditions, flight height or surface structures and might lead to signal alteration. The maximum distance between the parallel-flow and the across-flow radar traces is 11.7 m, so geometrical effects are assumed to be small but cannot be excluded entirely. Further uncertainties could be introduced from the time–to–depth conversion and the assumption of a spatially constant electromagnetic wave speed profile.

Table 1: **Comparison of travel-time analysis at six crosspoints near the East Greenland ice-core project (EGRIP).** The table shows the apparent horizontal anisotropy $\Delta\lambda_{\mathrm{a}}$ inferred from a regression through the origin, $\Delta\lambda_{\mathrm{a}}^{0}$, 200 m, $\Delta\lambda_{\mathrm{a}}^{200}$, and 500 m, $\Delta\lambda_{\mathrm{a}}^{500}$. δ is the angle between the two crossing flight lines and $\tilde{a}(\Delta\lambda_{\mathrm{a}})$ and $\tilde{s}(\Delta\lambda_{\mathrm{a}})$ are the mean and standard deviations of $\Delta\lambda$ inferred from the six crosspoints. The crosspoint locations are shown in Fig. 3b.

crosspoint (lat, lon)	$\Delta\lambda_{\mathrm{a}}^{0}$	$\Delta\lambda_{\mathrm{a}}^{200}$	$\Delta\lambda_{\mathrm{a}}^{500}$	δ
1 (75.6298,-35.9904)	0.435	0.448	0.488	89.9
2 (75.6296,-35.9895)	0.389	0.403	0.443	78.0
3 (75.6297,-35.9906)	0.492	0.508	0.556	86.1
4 (75.6296,-35.9891)	0.400	0.405	0.433	86.6
5 (75.6296,-35.9890)	0.505	0.515	0.546	89.7
6 (75.6295,-35.9892)	0.381	0.400	0.456	89.2
$\tilde{a}(\Delta\lambda)$	0.434	0.447	0.487	
$\tilde{s}(\Delta\lambda)$	0.054	0.053	0.053	

Table 2: **Example of travel-time analysis for crosspoint 3.** $t_{\hat{x}}$ and $t_{\hat{y}}$ are the travel times picked from reflections at depth z from $\hat{x}$- and $\hat{y}$-polarised radar traces, respectively. Δt is the travel-time difference ($t_{\hat{y}} - t_{\hat{x}}$). Superscript 0 indicates travel times and travel-time differences from a linear regression through the origin, superscript 200 m and 500 m denote the corresponding values for linear regression through 200 m and 500 m points respectively. $\overline{\varepsilon_{\hat{x}}}$, $\overline{\varepsilon_{\hat{y}}}$ and $\Delta\varepsilon_{\mathrm{a}}$ at the bottom of the table are the directional relative dielectric permittivities and apparent horizontal relative permittivity difference derived from the corresponding linear regression.

z [m]	$t_{\hat{x}}$[µs]	$t_{\hat{y}}$[µs]	Δt[µs]	$t_{\hat{x}}^{0}$[µs]	$t_{\hat{y}}^{0}$[µs]	Δt^{0}[µs]	$t_{\hat{x}}^{200}$[µs]	$t_{\hat{y}}^{200}$[µs]	Δt^{200}[µs]	$t_{\hat{x}}^{500}$[µs]	$t_{\hat{y}}^{500}$[µs]	Δt^{500}[µs]
0	-	-	-	0	0	0	-	-	-	-	-	-
200	-	-	-	-	-	-	2.347	2.353	0.0063	-	-	-
459.8	5.313	5.319	0.005	5.395	5.409	0.015	5.397	5.412	0.015	-	-	-
484.6	5.604	5.614	0.009	5.686	5.701	0.015	5.688	5.704	0.016	-	-	-
500	-	-	-	-	-	-	-	-	-	5.866	5.882	0.016
540.9	6.269	6.283	0.013	6.346	6.363	0.017	6.349	6.367	0.018	6.357	6.376	0.019
560	6.504	6.500	-0.004	6.570	6.588	0.018	6.573	6.591	0.018	6.582	6.602	0.020
709.3	8.272	8.267	-0.005	8.322	8.344	0.022	8.326	8.349	0.023	8.336	8.362	0.025
735.7	8.580	8.586	0.006	8.632	8.655	0.023	8.635	8.659	0.024	8.646	8.673	0.026
755.8	8.813	8.828	0.016	8.868	8.891	0.024	8.871	8.896	0.025	8.883	8.910	0.027
772.6	9.014	9.025	0.011	9.065	9.089	0.024	9.068	9.094	0.025	9.080	9.108	0.028
782.1	9.133	9.132	-0.001	9.176	9.201	0.025	9.180	9.206	0.026	9.192	9.220	0.028
807.5	9.425	9.441	0.016	9.474	9.500	0.026	9.478	9.505	0.026	9.490	9.519	0.029
828.3	9.674	9.683	0.009	9.718	9.744	0.026	9.722	9.749	0.027	9.735	9.764	0.030
851.9	9.949	9.968	0.019	9.995	10.022	0.027	9.999	10.027	0.028	10.012	10.043	0.030
931.7	10.889	10.917	0.028	10.931	10.961	0.030	10.936	10.966	0.030	10.950	10.983	0.033
981.8	11.481	11.511	0.030	11.519	11.550	0.031	11.524	11.556	0.032	11.539	11.574	0.035
1030.4	12.064	12.080	0.016	12.089	12.122	0.033	12.094	12.128	0.034	12.110	12.147	0.037
1165.2	13.648	13.688	0.039	13.671	13.708	0.037	13.677	13.715	0.038	13.694	13.736	0.042
1222.5	14.324	14.369	0.045	14.343	14.382	0.039	14.349	14.389	0.040	14.368	14.411	0.044
1916.8	22.525	22.608	0.082	22.489	22.550	0.061	22.499	22.561	0.063	22.528	22.596	0.069
2048.5	24.085	24.167	0.082	24.034	24.099	0.065	24.045	24.111	0.067	24.075	24.149	0.073
2166.1	25.475	25.562	0.087	25.414	25.483	0.069	25.425	25.496	0.071	25.458	25.535	0.077
2674.3	31.491	31.580	0.089	31.377	31.461	0.085	31.390	31.477	0.087	31.430	31.526	0.096
				$\overline{\varepsilon_{\hat{x}}}$	$\overline{\varepsilon_{\hat{y}}}$	$\Delta\varepsilon_{\mathrm{a}}$	$\overline{\varepsilon_{\hat{x}}}$	$\overline{\varepsilon_{\hat{y}}}$	$\Delta\varepsilon_{\mathrm{a}}$	$\overline{\varepsilon_{\hat{x}}}$	$\overline{\varepsilon_{\hat{y}}}$	$\Delta\varepsilon_{\mathrm{a}}$
				3.093	3.110	0.017	3.096	3.113	0.017	3.104	3.122	0.019

1.2 Beat-signature analysis

Fujita et al. (2006) [2] showed with synthetic results as well as observational data that distinct nodes and extinction features in the return power are generated for birefringent ice (i.e. a COF that results in bulk horizontal anisotropy) and anisotropic scattering as a function of depth. Of particular interest for our application is the appearance of the so-called co-polarisation node. In the case of purely birefringent ice without anisotropic scattering, this node is strongest (i.e. has the least backscatter power) for a co-polarised antenna orientation of 45° with respect to the principal component of the bulk COF and indicates a 90° periodicity with depth [2]. This concept has been used to determine the horizontal COF anisotropy from RES data in previous studies [11–13]. As explained above, the difference in the horizontal components of the bulk dielectric permittivities $\Delta\varepsilon$ defined in Equation (4) causes a continuous difference in two-way travel time, $\Delta t(z)$, of the two principle waves which can also be expressed as a phase difference $\phi(z) = 2\omega\Delta t(z)$. Factor 2 arises from the consideration of two-way travelling and $\omega = 2\pi f$ is the angular frequency of the radar wave with centre frequency f. The change of ϕ with depth gives rise to a continuous modulation of the backscattered power. In analogy to signal mixing, this can also be considered as a beat or modulation frequency f_{mod} from two superimposed waves at different frequencies, in our cases originating from the wave speed differences, as expressed in Equation (6). The beat-signature results in destructive interference, a so-called co-polarisation extinction node, when the phase difference ϕ between the ordinary and extraordinary wave component is an odd multiple of π [2]. Neglecting anisotropic scattering and approximating Equation (13) in Fujita *et al.* [2] with a Taylor expansion [14] results in

$$\phi(z) = \frac{4\pi f}{c_0}\int_z^0 \frac{\Delta\varepsilon(z)}{2\sqrt{\varepsilon_{\mathrm{xy}}}}\mathrm{d}z = \frac{2\pi f}{c_0}\Delta\varepsilon^{\mathrm{m}}\int_z^0 \frac{\Delta\lambda(z)}{\sqrt{\varepsilon_{\mathrm{xy}}}}\mathrm{d}z, \tag{11}$$

where $\varepsilon_{\mathrm{xy}}$ is the mean of the horizontal relative permittivities. Assuming a vertically constant COF with a constant eigenvalue difference $\Delta\lambda$ and thus permittivities, Equation (11) simplifies to

$$\phi(z) = \frac{2\pi f\Delta\varepsilon^{\mathrm{m}}}{c_0\sqrt{\varepsilon_{\mathrm{xy}}}}\Delta\lambda z. \tag{12}$$

Using the wave number, k_{mod}, of the beat signature to relate the phase difference, ϕ, to propagated depth, z, allows us to write

$$\phi(z) = 2k_{\mathrm{mod}}z = \frac{4\pi\sqrt{\varepsilon_{\mathrm{xy}}}f_{\mathrm{mod}}}{c_0}z. \tag{13}$$

Here, again, the first factor of 2 arises from the consideration of two-way propagation.

Setting Equation (13) equal to Equation (12) finally yields

$$\Delta\lambda = \frac{2\varepsilon_{\mathrm{xy}}}{\Delta\varepsilon^{\mathrm{m}}}\frac{f_{\mathrm{mod}}}{f}, \tag{14}$$

thus linearly connecting the horizontal anisotropy to the beat frequency, f_{mod}, and the centre frequency, f, of the radar system.

In Equation (14), we can also use the period $T_{\mathrm{mod}} = f_{\mathrm{mod}}^{-1}$ of the beat signal, e.g. directly from a radargram in the time domain and pick the time difference between neighbouring power extinction nodes (minima) to obtain

$$\Delta\lambda = \frac{2\varepsilon_{\mathrm{xy}}}{f\Delta\varepsilon^{\mathrm{m}}}\frac{1}{T_{\mathrm{mod}}}. \tag{15}$$

Minima in the beat signature are caused if the two waves are anti-phase, i.e. if they have a phase difference of odd multiples of π, $\Delta\phi = (2n+1)\pi$. Considering the beat signature as the image of a wave in the two-way travel-time domain (as we can see it in the radargram, see Fig. 4–6) with phase θ_{mod} we can write

$$\theta_{\mathrm{mod}} = k_{\mathrm{mod}}s = k_{\mathrm{mod}}\frac{c_0 t}{\sqrt{\varepsilon}}, \tag{16}$$

with $k_{\text{mod}} = \frac{2\pi}{l_{\text{mod}}}$ being the wave number and l_{mod} the wavelength of the beat signature. The second factor, s, is the propagated path which can be calculated from the two-way travel time, t. The travelled path, s, is twice the actual depth as plotted in a radargram, $s = 2z$, and therefore

$$\theta_{\text{mod}} = 2k_{\text{mod}}z. \tag{17}$$

In the simplest case of two neighbouring minima, separated by depth Δz, the phases θ_{mod} at these two minima are 2π apart, i.e. $\Delta\theta_{\text{mod}} = 2\pi$. Thus we can write

$$\Delta\theta_{\text{mod}} = 2\pi = 2k_{\text{mod}}\Delta z = 2\frac{2\pi}{l_{\text{mod}}}\Delta z, \tag{18}$$

which yields

$$l_{\text{mod}} = 2\Delta z, \tag{19}$$

i.e. the wavelength, l_{mod}, of the beat signature is twice the distance between two minima.

Young *et al.* [11] exploited this effect along shallow airborne radar profiles over the eastern shear margin of Thwaites Glacier to deduce the bulk anisotropy in the upper $\sim$1500 m of the ice sheet. We applied the same approach here to derive the vertical COF variations, as described in the following subsections.

Manual determination of horizontal anisotropy from beat signature

To investigate the possibility of depth-variable COF anisotropy (or to evaluate the limitations of the assumption of a depth-constant COF anisotropy), we employed the methods of Young *et al.* [11] over a representative subset of radargrams aligning both parallel and perpendicular to the ice-flow direction. For each radargram frame, the beat signature minima were manually traced following a two-dimensional convolution with window dimensions of 138×344 m^2 and 87×278 m^2 for the UHF 750 MHz and AWI-UWB 195 MHz radargrams, respectively. The resulting traces were then automatically aligned to and smoothed over the 'true' locations of the nearest beat signature minima using a Gaussian-weighted moving average algorithm with a 500 m window. Examples of two profiles perpendicular to ice flow and near the EGRIP drill site are shown in Fig. 4 (UHF system with 750 MHz) and Fig. 5 (AWI-UWB system with 195 MHz). The method, in principle, can also determine the variation of the COF with depth, averaging over the vertical distance between two minima.

Semi-automatic determination of horizontal anisotropy from beat frequency

Although the manual determination of the COF resolves vertical variations of the beat frequencies – i.e. the vertical distance between power minima – to derive the variation of the horizontal COF anisotropy, the applicability to large data sets is limited as it is a very time-consuming process. We, therefore, developed a semi-automated approach, which extracts the beat frequency f_{mod} from radargrams by spectrogram analysis. The AWI airborne UWB radargrams, which have been processed in the standard processing chain [see 3], were further processed by a bandpass filter (100–750 kHz), time cutting the upper section to remove the surface multiple and the lower section with the bed reflection and strong folds, and a second application of the same bandpass filter. The remaining part of the radargram contains backscatter variations mainly caused by internal layers, most of which are of volcanic origin [6] and the beat-frequency modulation of the backscattered power caused by horizontal anisotropy.

All pre-processed traces of a radargram were first concatenated to obtain one long time series. We used the MATLAB^{TM} *spectrum* function to calculate a continuous spectrum of the time series, using frequency limits of 100 to 750 kHz, default segmentation with 50 % overlap of neighbouring segments for spectral estimates. This results in a spectrogram of the radar return power in the range of 100–750 kHz along the radar profile. Depending on the location, the spectrograms clearly show modes originating from the beat frequency (Fig. 6). In a next step, we automatically extracted the frequency of those dominating modes as a function of position, which was then manually checked

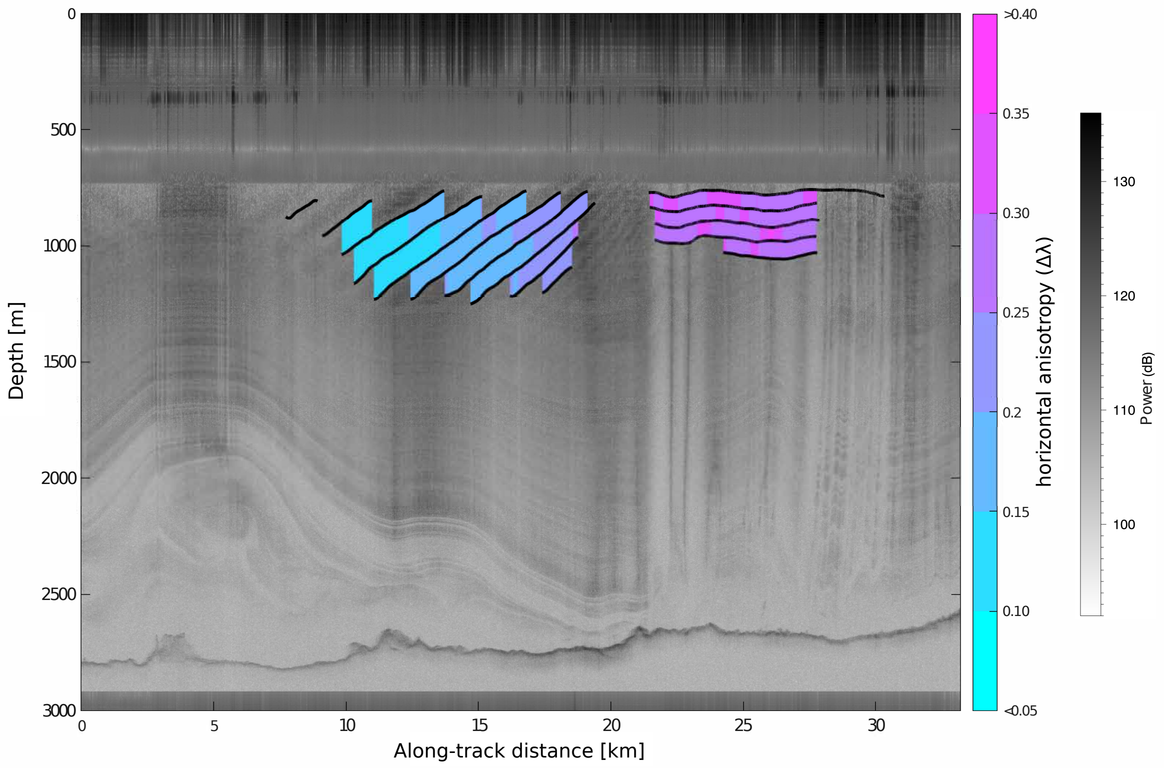

Figure 4: **Example of beat-signature analysis on ground-based radar profile.** The radargram was recorded with the Ultra High Frequency (UHF) Mills Cross system [15] with a centre frequency of 750 MHz. The profile runs from the East Greenland ice-core project (EGRIP, right) towards the north, perpendicular to ice flow and across the Northeast Greenland ice stream (NEGIS) shear margin, with a turn at the end (left). The signature shows an artefact in the upper 700 m from the source chirp, the bed reflection is visible around 2700–2800 m. Between 700 and 1200 m depth, beat signatures are highlighted. The horizontal crystal orientation fabric (COF) anisotropy is derived from the distance of neighbouring power extinction node lines and is indicated by colour, using the approach of Young *et al.* [11] .

for consistency and corrected for artefacts by correcting or removing wrong automatically determined modes (see red line in the bottom panel of Fig. 6). Finally, we obtained a spatial distribution of the dominating beat frequency, which was converted to $\Delta\lambda$ by Equation (14) (see Fig. 6 for an example). The approach allows to process and analyse the radargrams automatically, thus reducing the required time. We estimate the uncertainty in $\Delta\lambda$ to be around 0.05, i.e. 5 % of its theoretical maximum. However, the limitations are in parts ambiguities in the spectral analysis to determine the dominating mode, for instance, if internal layers are spaced at a vertical distance comparable to the beat signature. The approach is, therefore, most applicable in regions where the horizontal anisotropy is spatially changing, i.e. along radar profiles perpendicular to the flow field and across the shear margins. The results are best suited for the interpretation of the overall spatial distribution of the anisotropy rather than depicting the COF at a particular location without taking the spatial distribution into account.

Forward modelling of birefringence induced anisotropy

To demonstrate the effect of modelled polarimetric radio signal propagation through an ice column, we used the Fujita *et al.* [2] matrix-based model for a frequency of $f = 195$ MHz. Each model case

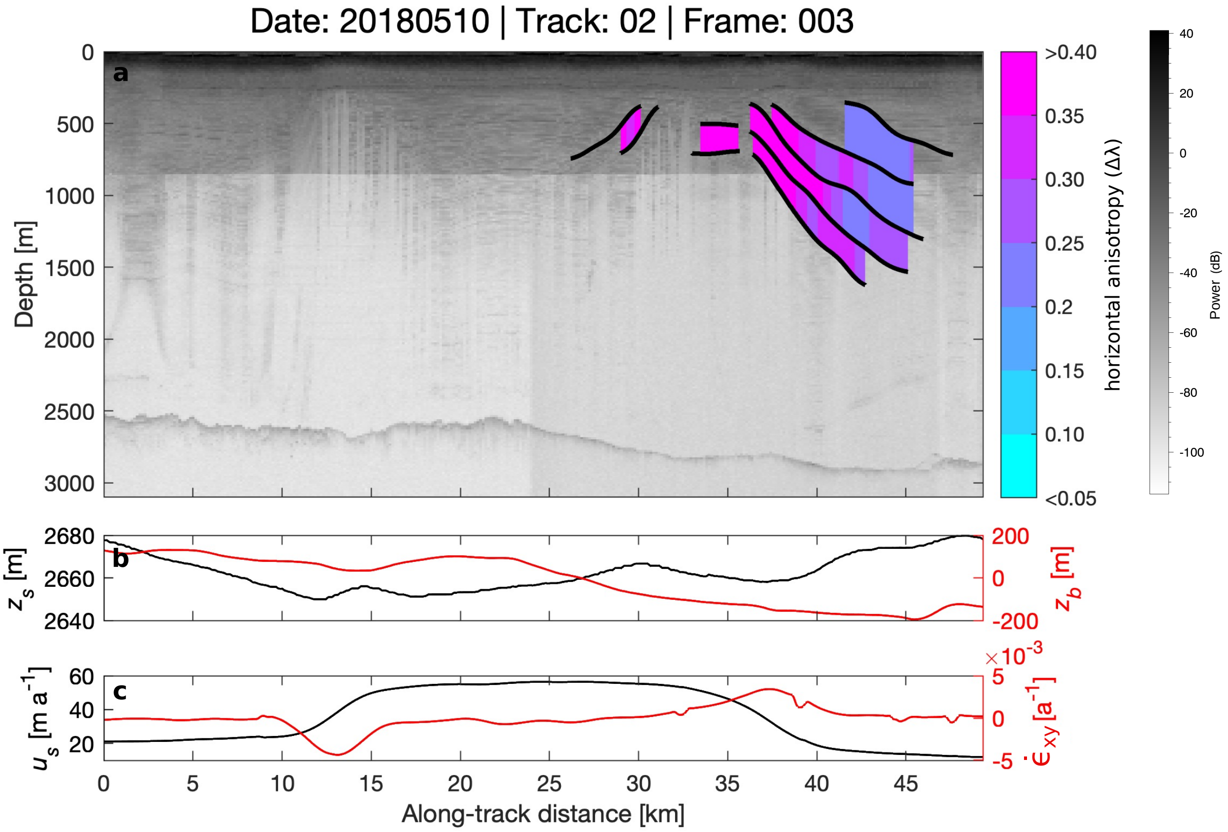

Figure 5: **Example of beat-frequency analysis on airborne profile. a** The radargram was recorded with an Ultra Wide Band (UWB) radar system with a centre frequency of 195 MHz [3]. The profile crosses NEGIS, with the East Greenland ice-core project (EGRIP) in the centre, from the southeast (left) to the northwest (right), perpendicular to ice flow and across both shear margins (visible around 13 km and 36 km along-track distance). The part of this profile northwest of EGRIP is close to the ground-based radar profile shown in Figure 4. The horizontal crystal orientation fabric (COF) anisotropy derived from the distance of neighbouring node lines is again indicated by colour, using the approach of Young *et al.* [11]. Panel **b** indicates surface, z_s, (black) and bed, z_b, (red) elevation along the radargram transect from BedMachine. Panel **c** shows the surface velocity, u_s, (black) and lateral shear strain $\dot{\epsilon}_{xy}$ (red) along radar transect derived from satellite-based surface velocities [16].

in Fig. 7 shows the effect of a slightly different horizontal anisotropy within the ice column defined by increasing the difference between the two horizontal eigenvalues ($\Delta\lambda$). The other parameters in the models, including the anisotropic scattering (set to zero) and COF orientation, remained constant. More details about the features in the forward model and the effect of other parameters are explained in Ershadi *et al.* [12].

1.3 Travel-time analysis of polarimetric phase-sensitive radio-echo-sounding (pRES) measurements

In general, the analysis of the horizontal COF anisotropy from the polarimetric pRES measurements follows the method described in Section 1.1 and in Zeising *et al.* [17]. First, we calculated the vertical travel-time difference between the HH- and the VV-polarised measurements from the cross-correlation of the amplitude- and the phase profiles. This method is widely used to derive vertical strain from repeated pRES measurements (e.g. Stewart *et al.* [18], Vaňková *et al.* [19], Zeising & Humbert [20], and Stewart [21]). Before calculating the cross-correlation, we applied the time-depth conversion and divided the HH-polarised measurement into segments of 6 m depth with 3 m overlaps, starting at a depth of 20 m. The cross-correlation between the HH- and the VV-polarised measurements were then calculated for each segment. A first coarse range difference

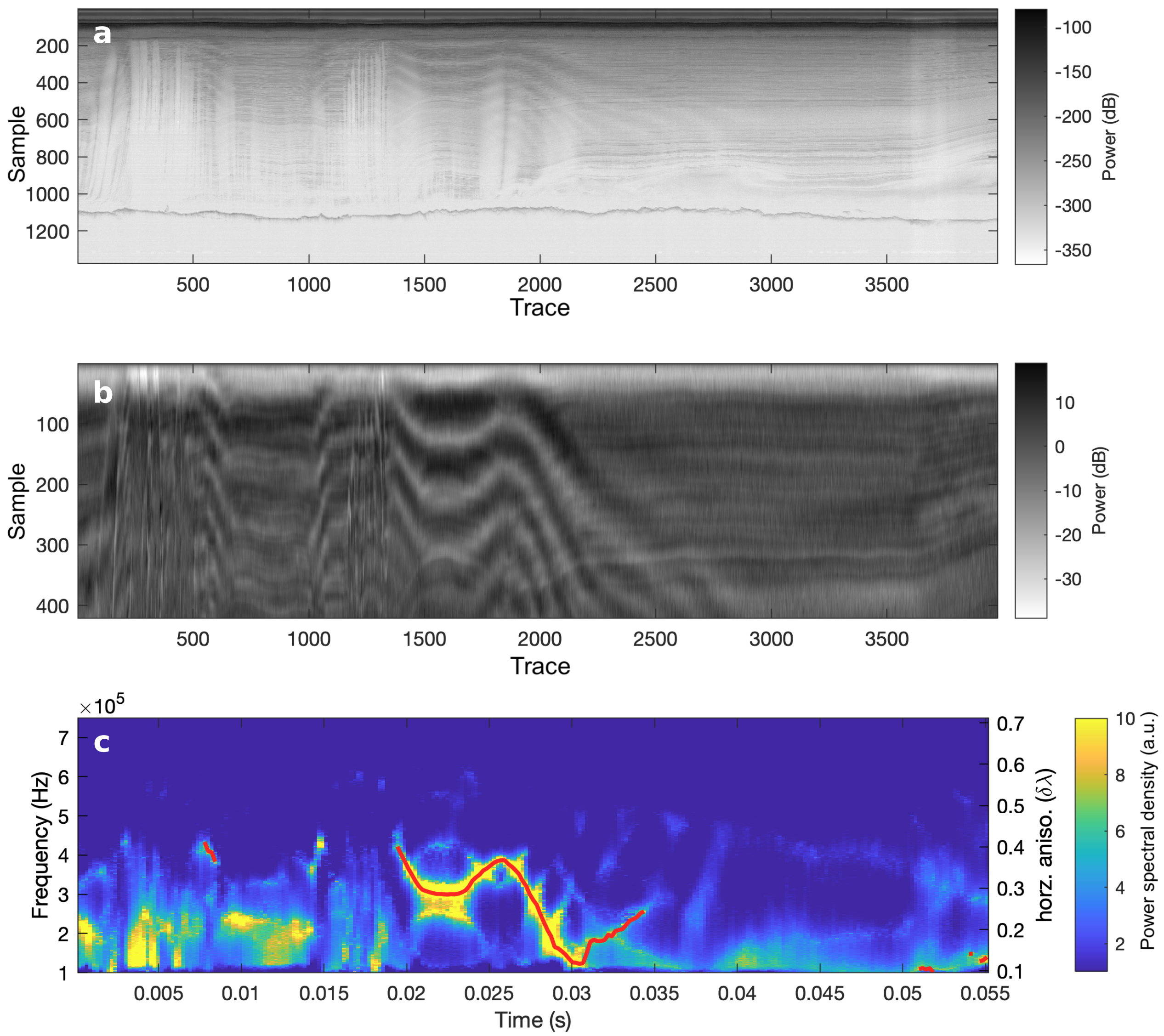

Figure 6: **Example of spectrogram analysis for Ultra Wide Band (UWB) profile 20180512-02-006. a** Original radargram. **b** Filtered and cropped radargram. **c** Spectrogram showing the relative power spectral density in colour from which we obtain the (manually checked and corrected) horizontal distribution of the beat frequency f_{mod} (red line) used in Equation (14) to calculate the horizontal anisotropy.

with a resolution of > 5 cm was derived from the lag with the largest amplitude-correlation value for segments with a correlation coefficient higher than 0.8. However, a low signal-to-noise level prevents the estimation of the coarse-range difference from a depth of roughly 1500 m and deeper.

In order to derive the fine-range difference with millimetre resolution, we used the phase shifts from the cross-correlation of the phase profiles. Here, we manually tracked the vertical distribution of the minimum phase shift nearby the coarse-range differences. Next, we derived the vertical distribution of the range difference (Δz) and the corresponding travel-time difference (Δt) from the selected lag and phase shift of each segment.

In contrast to the travel-time analysis at crosspoints of the airborne radar survey, the high vertical resolution of Δt obtained from pRES allows the estimation of the depth-varying relative permittivity. To do that, we determined the slopes $m_{\hat{x}}(z)$ and $m_{\hat{y}}(z)$ (see Equation (8)) as a function of depth by calculating the average slopes of the travel-time difference in a 200 m moving window, after smoothing with a 24 m moving median. Finally, we derived the difference in horizontal dielectric anisotropy and the difference in horizontal eigenvalues from Equation (9) and Equation (10). An example from the measuring site 31 km upstream of EGRIP is shown in Fig. 8.

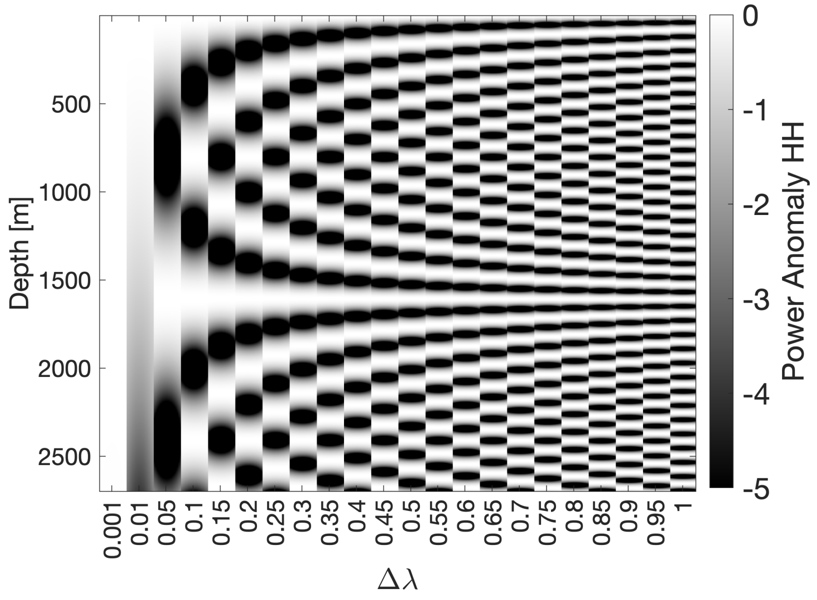

Figure 7: **Synthetic calculation of power anomaly for HH polarisation in a horizontally anisotropic medium.** $\Delta\lambda$ ranges from 0.001 to 1 (x-axis) and the ice thickness (y-axis) is assumed to be 2700 m. Each of the 22 columns shows the power anomaly for a rotation of the polarisation from 81° to 100°. The extinction nodes caused by birefringence correspond to the black nodes (i.e. lower power). Connecting nodes for a fixed polarisation would yield the extinction node lines in radargrams (e.g. Fig. 4–6). For very low anisotropy ($\Delta\lambda = 0.001$), no node is present. For $\Delta\lambda = 0.01$, the first node extends over the full ice column, for $\Delta\lambda = 0.05$ two nodes appear, etc. The calculations were done with a matrix-based model [2, 14], closely following the approach by Ershadi *et al.* [12].

1.4 Methods comparison

Figure 9 shows the $\Delta\lambda$ values obtained by different methods versus distance from EGRIP along a profile crossing the ice stream. The results obtained from Elmer/Ice (for details see Section 2.1) and the ice-core measurements at EGRIP and S5 show the absolute horizontal anisotropy. The results from radar-derived methods (pRES, beat signature and crosspoint travel-time analysis) should be considered as lower-bound estimates due to ambiguities related to the orientation of radar-wave polarisation relative to the COF, and the visibility of the beat-signature which can be obscured by decreased return power, e.g. through heavy folding, or internal stratigraphy. The horizontal anisotropy from the EGRIP ice core is representative over a depth range between 500–2200 m while the S5 data point is based on only one depth sample at 68 m. The modelling results from Elmer/Ice are depth-averages across the entire ice column. Crosspoint travel-time analysis are depth-averages across the picked reflections down to a depth of ~2600 m, although this varies between crosspoints as the deepest reflections were not clearly visible everywhere. Finally, the depth-average $\Delta\lambda$ from the beat-signature and pRES travel-time analysis are representative across the top 1700 m and 1500 m, respectively.

All methods consistently show low horizontal anisotropies ($\Delta\lambda \sim$ 0–0.3) outside the ice stream. Elmer/Ice shows higher horizontal anisotropy in the northwestern shear margin than the southeastern one, while the shear margin core, S5, shows an eigenvalue difference of 0.8, similar to what is obtained by Elmer/Ice in the northwestern margin. An increasing eigenvalue difference from outside the ice stream towards the shear margin is also observed in the radar-derived methods, though the values are smaller due to the reasons outlined above. Inside the ice stream, strong horizontal anisotropies between 0.4–0.7 are obtained by Elmer/Ice, pRES, travel-time analysis at radar crosspoints and confirmed by the EGRIP ice core, while the beat-signature underestimates the horizontal anisotropy in this area.

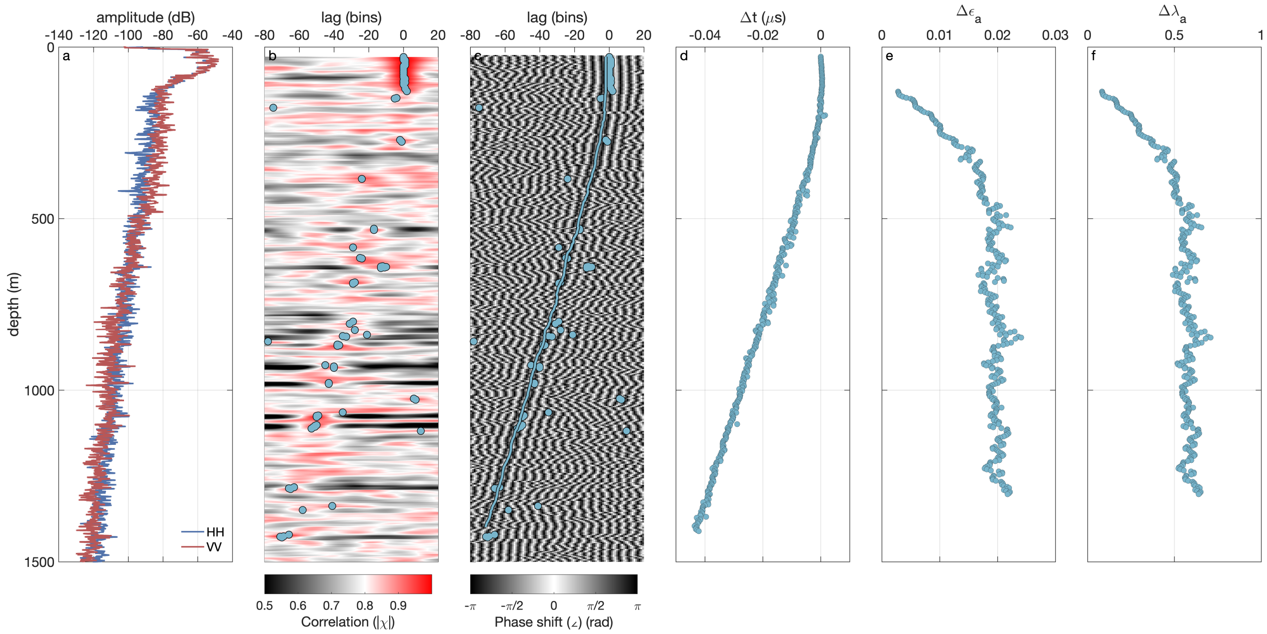

Figure 8: **Analysis of the difference in horizontal eigenvalues from polarimetric phase-sensitive radio-echo-sounding (pRES) measurements.** These pRES measurements were performed in the ice stream centre, 31 km upstream of the East Greenland ice-core project (EGRIP). **a** Comparison of amplitude profiles of HH- (blue line) and VV-polarised (red line) measurement as a function of depth. **b** Cross-correlation ($|\chi|$) of both measurements as a function of lag and depth. Blue dots mark the lag of highest correlation of the segment for those exceeding the correlation value of 0.8. **c** Phase shift as a function of lag and depth. The blue dots are the same as in panel b. The blue line marks the tracked minimum phase shift. **d** Difference in two-way travel time between both measurements at the same depth. **e** Difference in horizontal dielectric anisotropy $\Delta\varepsilon_{\mathrm{a}}$. **f** Difference in horizontal eigenvalues $\Delta\lambda_{\mathrm{a}}$.

2 Crystal orientation fabric evolution models

We used two COF evolution models to produce a more spatially complete map of COF that can be compared to measurements: the full-Stokes ice-flow model Elmer/Ice and the COF-evolution model Specfab which is decoupled from ice-dynamics processes.

2.1 Elmer/Ice

Elmer/Ice is an open source, full-Stokes ice-flow model using the finite element method. It solves the combined problems of ice flow, heat flow and COF evolution, thus accounting for both, the COF evolution due to temperature and deformation as well as the effect of the COF on the mechanical properties of ice.

Model equations

Ice flow is described by the incompressible Stokes equations:

$$\nabla \cdot \mathbf{u} = 0, \tag{20}$$

where $\mathbf{u}$ is the velocity, and

$$\nabla \cdot \boldsymbol{\sigma} + \rho_{\mathrm{i}}\mathbf{g} = 0, \tag{21}$$

where $\boldsymbol{\sigma}$ is the Cauchy stress tensor, ρ_{i} the density of ice, and $\mathbf{g}$ the force of gravity. These equations are linked using a nonlinear extension to the General Orthotropic Linear Flow Law [22] that, by

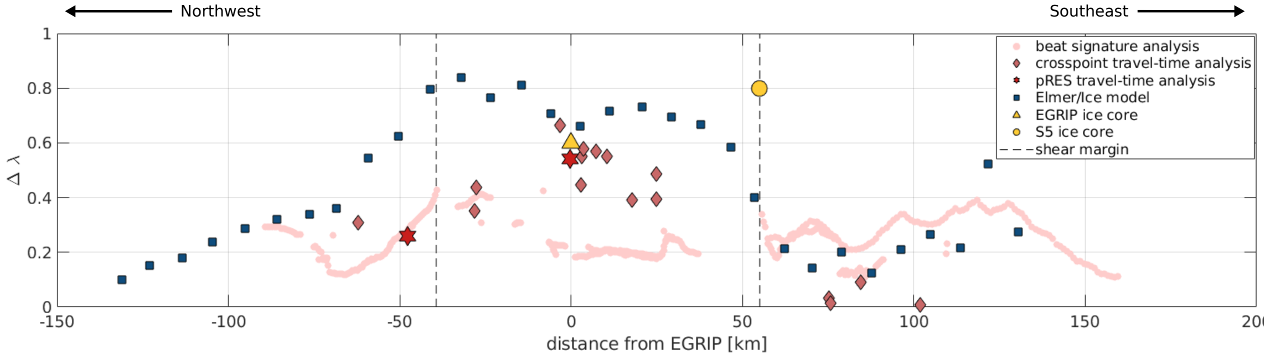

Figure 9: **Method comparison of horizontal anisotropy across the Northeast Greenland ice stream (NEGIS).** $\Delta\lambda$ obtained by different methods are compared on a profile crossing NEGIS near the East Greenland ice-core project (EGRIP). The left side of the figure corresponds to the northwestern part of the profile, the right side corresponds to southeast. Absolute horizontal anisotropy values are given by ice core measurements (EGRIP, S5; yellow markers) and the Elmer/Ice model (blue squares), while radar-based travel-time (crosspoints, pink diamonds; phase-sensitive radio-echo-sounding (pRES), red stars) and beat-signature analyses (pink dots) results indicate the apparent horizontal anisotropy, i.e. a lower-bound estimate.

analogy to Glen's flow law, introduces nonlinearity through the second invariant of the deviatoric stress tensor [23]:

$$\boldsymbol{\tau} = \eta_0 \sum_{r=1}^{3} \eta_r \mathrm{tr}(\mathbf{M}_r \cdot \dot{\boldsymbol{\epsilon}})\mathrm{dev}(\mathbf{M}_r) + \eta_{r+3}\mathrm{dev}(\dot{\boldsymbol{\epsilon}} \cdot \mathbf{M}_r + \mathbf{M}_r \cdot \dot{\boldsymbol{\epsilon}}) \tag{22}$$

where $\eta_{1...6}(\mathbf{a}^{(2)})$ are six dimensionless viscosities as a function of the COF, tr denotes trace, dev denotes the deviatoric portion of a tensor, $\mathbf{M}_{1...3}$ are the structure tensors that are the dyadic products of the COF's symmetry axes (i.e. the eigenvectors of $\mathbf{a}^{(2)}$), and $\eta_0 = (\tau_\mathrm{e}^{1-n})/2A(T)$, with τ_e being the second invariant of $\mathrm{dev}(\boldsymbol{\sigma})$. $A(T)$ is the temperature-dependent prefactor from Glen's flow law, and n is the flow-law exponent (taken to be 3). The viscosities $\eta_{1...6}(\mathbf{a}^{(2)})$ were found using a visco-plastic self-consistent model [24], assuming that ice with a single maximum COF deforms 10 times more quickly than isotropic ice given equal shear stress while it compresses 2.5 times more slowly along the direction of the principal COF axis.

The model was solved along flow tubes, so Equation (20) and Equation (21) are modified to account for a parameterised, flow-transverse coordinate $\tilde{y}$. In a coordinate system with $\tilde{x}$ along flow and z vertical,

$$\frac{\partial \sigma_{\tilde{x}\tilde{x}}}{\partial \tilde{x}} + \frac{\partial \sigma_{\tilde{x}z}}{\partial z} + \frac{\sigma_{\tilde{x}\tilde{x}} - \sigma_{\tilde{y}\tilde{y}}}{W(\tilde{x})}\frac{\partial W(\tilde{x})}{\partial \tilde{x}} = 0 \tag{23}$$

and

$$\frac{\partial \sigma_{\tilde{x}z}}{\partial \tilde{x}} + \frac{\partial \sigma_{zz}}{\partial z} + \frac{\sigma_{\tilde{x}z}}{W(\tilde{x})}\frac{\partial W(\tilde{x})}{\partial \tilde{x}} = \rho_\mathrm{i}\mathbf{g} \tag{24}$$

for a flow tube of width $W(\tilde{x})$ (e.g. Hvidberg [25]).

COF evolution was assumed to occur solely by lattice rotation (while neglecting recrystallisation) so that

$$\frac{d\mathbf{a}^{(2)}}{dt} = \mathbf{W} \cdot \mathbf{a}^{(2)} - \mathbf{a}^{(2)} \cdot \mathbf{W} - (\mathbf{C} \cdot \mathbf{a}^{(2)} + \mathbf{a}^{(2)} \cdot \mathbf{C}) + 2\mathbf{a}^{(4)} : \mathbf{C} + \mathbf{D} \tag{25}$$

where $\mathbf{W}$ is the spin tensor (the anti-symmetric portion of the deformation), : is the double inner product, $\mathbf{D}$ is used for regularisation, and

$$\mathbf{C} = (1-\alpha)\dot{\boldsymbol{\epsilon}} + \alpha k_\mathrm{s} A \tau_\mathrm{e}^{n-1}\boldsymbol{\tau} \tag{26}$$

where α is an interaction parameter controlling the relative importance of bulk stress and strain in causing COF evolution, here taken to be $\alpha = 0.06$ [23] and k_s is the enhancement of shear for

a single maximum fabric relative to isotropy, taken to be 10. The term $2\mathbf{a}^{(4)} : \mathbf{C}$ in Equation (25) is approximated using an invariant-based fit to a parameterised ODF to get $\mathbf{a}^{(4)}$ as a function of $\mathbf{a}^{(2)}$ [26, 27]. We used the regularisation

$$\mathbf{D}\left(\mathbf{a}^{(2)}\right) = \begin{cases} \xi e^{\frac{\ln(10)T}{10}}(1 - 3a_{ij}^{(2)})||\mathbf{C}||_2 & \text{if } i = j \\ \xi e^{\frac{\ln(10)T}{10}}(-3a_{ij}^{(2)})||\mathbf{C}||_2 & \text{if } i \neq j \end{cases}, \tag{27}$$

with $\xi = 2.0 \times 10^{-3}$.

The temperature was determined using the standard advection-diffusion equation,

$$\rho_{\mathrm{i}} q \left(\frac{\partial T}{\partial t} + \mathbf{u} \cdot \nabla T\right) = \nabla \cdot (\kappa \nabla T) + \Psi, \tag{28}$$

where q and κ are the heat capacity and heat conductivity of ice, respectively, and $\Psi = \boldsymbol{\sigma} : \dot{\boldsymbol{\epsilon}}$ is the strain heating.

Horizontal shear is large in much of the NEGIS catchment and occurs out of the plane of the 2.5-D model used here. $\dot{\epsilon}_{\tilde{x}\tilde{y}}$ was therefore parameterised in the COF evolution equations. The portion of horizontal shear experienced in the model plane is

$$\dot{\epsilon}_{\tilde{x}\tilde{y}} = \frac{1}{2}\frac{\partial u_{\mathrm{x}}}{\partial \tilde{y}} - \frac{u_{\mathrm{x}}}{2r}, \tag{29}$$

where r is the radius of curvature of the flow line. We assumed that the intensity of shear at depth scales with the along-flow velocity so

$$\frac{\partial u_{\mathrm{x}}}{\partial \tilde{y}} \approx \frac{\partial u_{x,\mathrm{s}}}{\partial \tilde{y}} \frac{u_{\mathrm{x}}(z)}{u_{x,\mathrm{s}}}, \tag{30}$$

where $u_{x,\mathrm{s}}$ is the surface velocity. Combining Equation (29) and Equation (30), the horizontal shear strain rate in model coordinates can be approximated as

$$\dot{\epsilon}_{\tilde{x}\tilde{y}}(z) \simeq \frac{1}{2}\left(\frac{\partial u_{x,\mathrm{s}}}{\partial \tilde{y}} \frac{u_{\mathrm{x}}(z)}{u_{x,\mathrm{s}}} - \frac{u_{\mathrm{x}}(z)}{r}\right). \tag{31}$$

We neglected the contribution of $\dot{\epsilon}_{\tilde{x}z}$ to the COF evolution since that term can be shown to be small [28]. In addition, we neglected the effect of horizontal shear upon the effective viscosity but included it in the temperature evolution. Before each simulation, we calculated r and $\frac{\partial u_{x,s}}{\partial \tilde{y}}$ for each model domain from a multi-year InSAR composite [29], smoothed to 2-km resolution, and kept them fixed throughout the model runs.

Boundary Conditions

At the surface, we simply imposed zero normal stress. The accumulation rate was taken to match the 1970–2016 mean from RACMO 2.3 [31]. At the basal boundary, we used the pattern of basal melt inferred from radar and 1-D modelling from MacGregor *et al.* [32], with freeze-on zeroed out. In addition, we scaled the overall pattern of basal mass loss in order to achieve mass balance (requiring a reduction of up to 50 % compared to the published values), so that final ice thicknesses remain realistic.

At the downstream end of the domain, within the ice stream, we imposed that the horizontal velocity matches the surface velocity at all depths, i.e. we forced all motion to take place via sliding. We expect significant sensitivity to this assumption within a few ice thicknesses of the boundary, so results near the downstream end of the model domain must be treated accordingly.

When motion is purely the result of sliding, Equation (20) implies that the vertical strain rate is constant, so the vertical velocity varies linearly with depth. We assumed that the downstream boundary is at its steady state thickness, so imposed that the vertical velocity varies linearly from the accumulation rate at the surface to the melt rate at the bed.

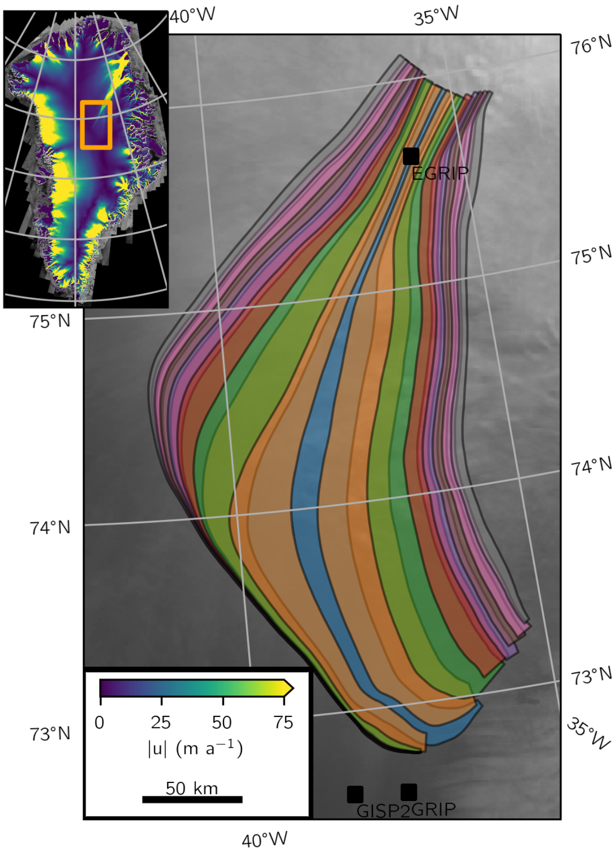

Figure 10: **Model domains of the Elmer/Ice flow model.** Every other domain across flow is plotted. Background from Joughin *et al.* [30]. Inset shows location in Greenland, plotted atop ice-flow velocities [29].

At the inflow boundary, we imposed a depth-variable horizontal velocity scaled to match observations at the surface [29]. Assuming that ice is isothermal, isotropic, and flows purely by internal deformation, the velocity can be shown to follow [33]:

$$\mathbf{u}_{\text{in}}(z) = \mathbf{u}_{\text{s,in}} \left[1 - \left(\frac{H_{\text{in}} - z}{H_{\text{in}}} \right)^{n+1} \right], \tag{32}$$

where H_{in} is the ice thickness at inflow, and we took $n = 4$ as an ad-hoc fit to the weakening from COF and temperature found at depth within the model domain at $n = 3$.

To reasonably match observed surface velocities, the model requires a variable basal velocity. We imposed a spatially variable basal drag with the results of inverse modelling using a 3-dimensional model of the upper catchment of NEGIS, determined in the same manner as Holschuh *et al.* [34] but updated to suit the region of interest here. We used a linear Weertman sliding law, i.e. $\tau_b = Bu^m$, where $m = 1$ and B is determined from the inverse results. In the final 2 km of the domain, the inversion-determined B was linearly tapered to zero to ensure a smooth transition to the plug-flow boundary condition we used there.

We assumed that the surface ice is isotropic, while at the upstream end, we imposed the profile from the NGRIP ice core [35]. However, the results are insensitive to this choice since the memory of this condition is erased before the ice reaches the NEGIS onset region. For temperature boundary conditions, the surface was held at the 1970–2013 2-m temperature from RACMO 2.3 [31] and the geothermal heat flux at the base was taken from a global gridded product [36].

2.2 Downstream crystal orientation fabric (COF) evolution modelling with Specfab

Radar-inferred horizontal anisotropies over NEGIS decrease downstream of EGRIP. The model domain of our Elmer/Ice flow model simulations extends, however, only approximately 40 km down-

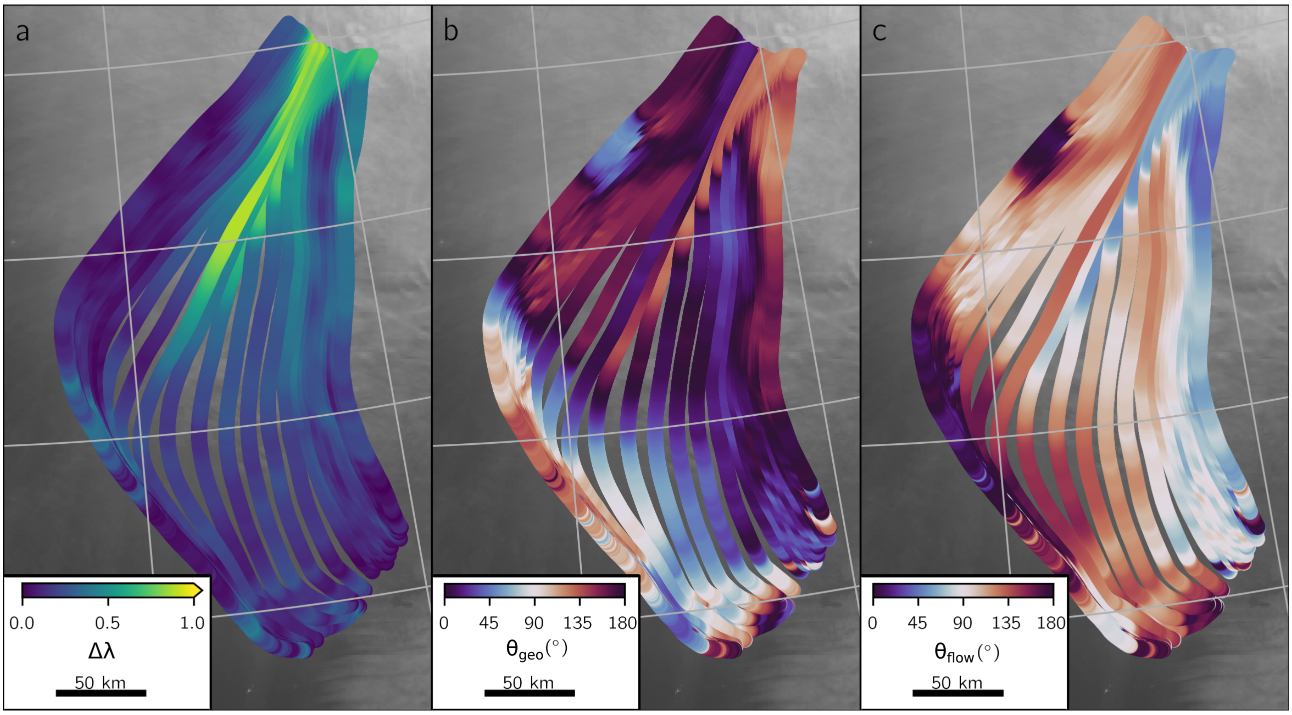

Figure 11: **Results from anisotropic flow modelling with Elmer/Ice.** We define the coordinate system to have flow at angle β counterclockwise from North Polar Stereographic east, and the largest crystal orientation fabric (COF) eigenvalue at θ_{geo} (see Fig. 1). Note that for the COF, angles are equivalent mod(180°), since c-axes do not have a direction, and we choose to define them to be in the range $0° \leq \theta_{\text{geo}} \leq 180°$. **a** Horizontal eigenvalue difference, $\Delta\lambda$. **b** The rotation of the largest horizontal eigenvector from Polar Stereographic East. **c** The rotation of the largest horizontal eigenvector from model $\tilde{x}$ (i.e. flow direction).

stream of EGRIP due to loss of numerical stability associated with higher ice flow velocities. Hence, the COF evolution in that area cannot be validated with Elmer/Ice. Instead, we used the spectral COF model for polycrystalline materials by Rathmann *et al.* [37] to simulate the COF evolution of an ice parcel (large enough to statistically represent the COF) along the flow line starting at EGRIP. This model is identical to the lattice rotation model in Elmer/Ice, but takes $\alpha = 0$ and is posed in spectral space instead of tensorial space. The used regularisation is therefore slightly different (spectrally sharpened to disproportionately affect highest wave-number modes), although also based on a diffusion process [37, 38]. Regularisation was, like in Elmer/Ice, taken to be proportional to the bulk-strain rate magnitude such that COF evolution depends only on the total accumulated strain, and not the rate at which the total strain was reached. Separate 3-D cube-crushing experiments were also performed in Elmer/Ice to compare the two models, finding an excellent agreement for various modes of deformation (not shown; soon to be published elsewhere by D. Lilien). Overall, the effect of regularisation can therefore be regarded as minimal in our Specfab simulations.

We consider an ice parcel seeded at EGRIP with a girdle-type COF that matches the average COF measured in the ice-core [39],

$$\mathbf{a}^{(2)} = \begin{pmatrix} 0 & 0 & 0 \\ 0 & 0.6 & 0 \\ 0 & 0 & 0.4 \end{pmatrix}, \tag{33}$$

and let it travel downstream along a flow line derived by the MeASUREs multi-year surface velocity field [29]. The flow line was divided into segments of distance travelled in one year. For each segment, the strain-rate tensor, $\dot{\boldsymbol{\epsilon}}$, was derived from surface velocities in a curvilinear coordinate system

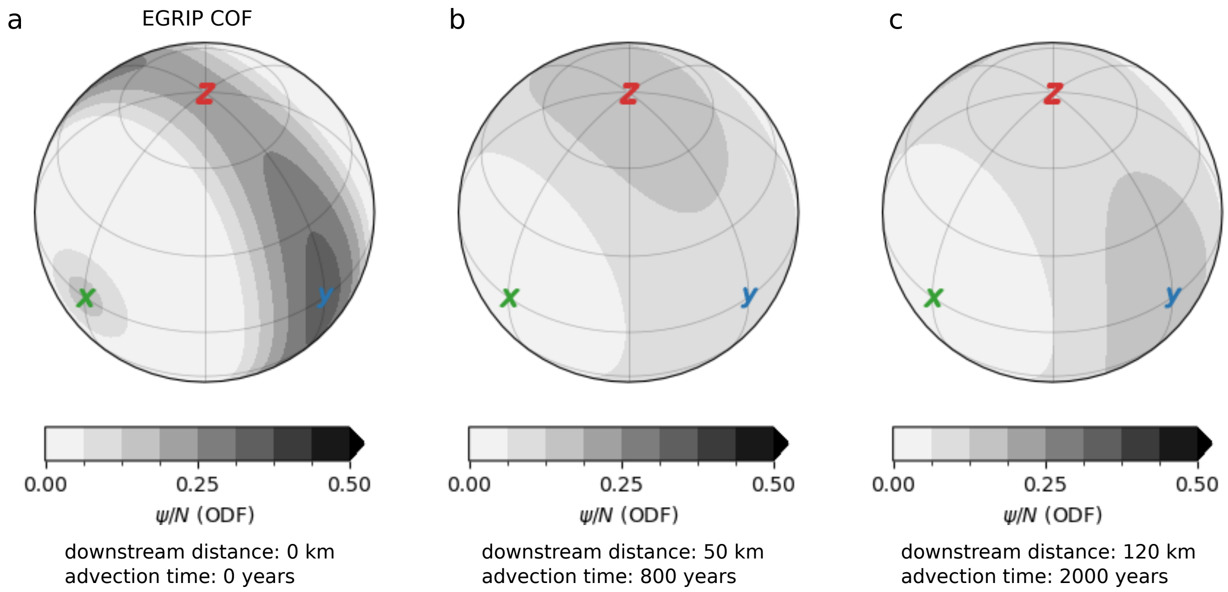

Figure 12: **Crystal orientation fabric (COF) evolution along a downstream flow line starting at the East Greenland ice-core project (EGRIP).** The EGRIP-type girdle in **a** transforms into a vertical single maximum in **b** within a distance of 50 km, corresponding to approximately 800 years. Further deformation leads to a weak vertical girdle at the end of the flow line as shown in **c**, corresponding to 120 km downstream of EGRIP or an advection time of 2000 years. The COF was calculated using the 'Specfab' model developed by Rathmann *et al.* [37], with spin and strain-rate tensors derived from satellite-based surface velocities [29]. The COF is shown as orientation distribution function (ODF) of the c-axes where x points in flow-direction and z is vertical.

following the flow line under an assumption of negligible vertical shear and incompressibility:

$$\dot{\epsilon}_{\tilde{x}\tilde{x}} = \frac{\partial u_x}{\partial \tilde{x}}, \quad \dot{\epsilon}_{\tilde{y}\tilde{y}} = \frac{\partial u_y}{\partial \tilde{y}}, \quad \dot{\epsilon}_{\tilde{z}\tilde{z}} = -\dot{\epsilon}_{\tilde{x}\tilde{x}} - \dot{\epsilon}_{\tilde{y}\tilde{y}}, \tag{34}$$

$$\dot{\epsilon}_{\tilde{x}\tilde{y}} = \dot{\epsilon}_{\tilde{y}\tilde{x}} = \frac{1}{2}\left(\frac{\partial u_x}{\partial \tilde{y}} + \frac{\partial u_y}{\partial \tilde{x}}\right) \quad \dot{\epsilon}_{\tilde{x}z} = \dot{\epsilon}_{\tilde{y}z} = 0. \tag{35}$$

The spin tensor, **W**, was calculated from the surface velocities, assuming the vertical components are zero:

$$\mathbf{W} = \frac{1}{2}\left(\tilde{\nabla}\mathbf{u} + (\tilde{\nabla}\mathbf{u})^{\mathrm{T}}\right) = \mathbf{0}. \tag{36}$$

For each one-year time step, the Lagrangian update to the parcel's COF is given by the strain-rate and spin tensors over the traversed flow-line segment. The end of the flow line corresponds to 2000 years advection time from EGRIP. Figure 12 shows snapshots of the corresponding normalised c-axes distribution (orientation distribution function) at three points along the flow line. The initial girdle is found to slowly evolve into a vertical single maximum at a distance of approximately 50 km or a time period of 800 years. With continuing deformation, the COF transitions into a vertical girdle which persists from 100 km downstream of EGRIP (corresponding to an advection time of 1720 years) until the end of the flow line 120 km downstream. Figure 13 shows the cumulative strain and COF eigenvalues. The positive strain (extension) perpendicular to the flow direction leads to the rotation of c-axes away from the flow-transverse (y) direction into the vertical direction of dominant compression. In the second half of the flow line, this process is reversed, as the ice-flow acceleration causes positive (extensive) along-flow strain, while the extension in the flow-transverse direction stops. Consequently, the c-axes tend to rotate back into a girdle-type COF configuration, although in a weaker form than at EGRIP.

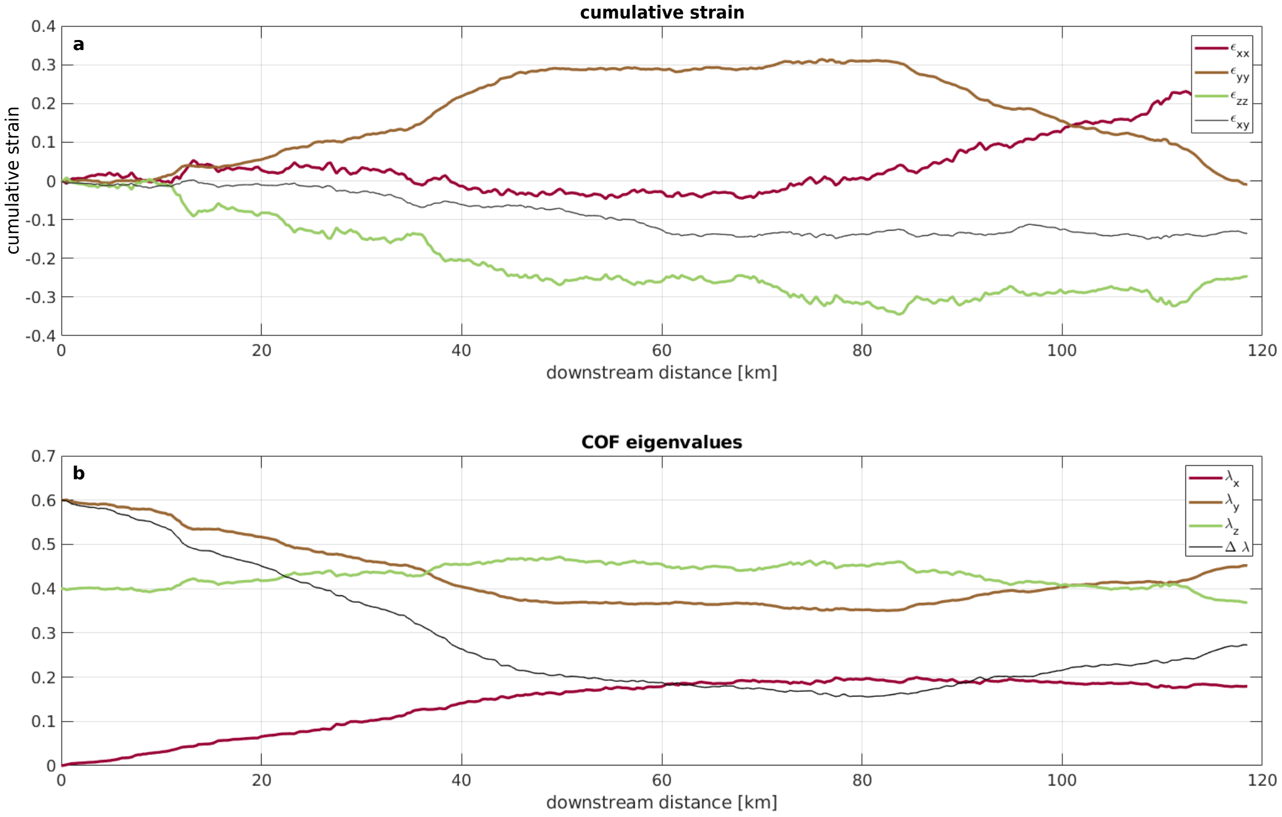

Figure 13: **Comparison of cumulative strain and fabric evolution.** **a** Cumulative strain along a 120 km long flow line starting at the East Greenland ice-core project (EGRIP) where ϵ_{xx}, ϵ_{yy} and ϵ_{zz} are the flow-parallel, flow-transverse and vertical strain respectively, and ϵ_{xy} is horizontal shearing. The cumulative strain was calculated from the strain-rate tensors of yearly intervals along the flow line derived from surface velocities [29]. Positive/negative strain corresponds to extension/compression. **b** Evolution of crystal orientation fabric (COF) eigenvalues (λ_x,λ_y,λ_z) and the horizontal eigenvalue difference ($\Delta\lambda = \lambda_y - \lambda_x$) calculated with the Specfab model [37].

We emphasise, however, that the COF evolution model is a linear partial differential equation. As such, any unaccounted, superimposed mode of deformation would lead to a corresponding superimposed imprint on the modelled c-axis distributions. For example, if vertical simple shear is present (not modelled here), a superimposed vertical single-maximum is to be expected that has little-to-no horizontal anisotropy. Such vertically symmetric contributions to the total COF pattern are, however, not detectable by our radar methodology which requires horizontal anisotropy to be present.

3 Flow enhancement factors

To evaluate the effect that the inferred COF has upon ice flow, we calculated the bulk directional enhancement factors following Rathmann *et al.* [37] and Rathmann & Lilien [38], defined as the anisotropic-to-isotropic strain-rate ratio [40]:

$$E_{vw} = \frac{\dot{\epsilon}_{vw}}{\dot{\epsilon}_{vw}^{iso}}, \tag{37}$$

where subscript vw indicates the component (direction) of interest, and $\dot{\epsilon}_{vw}$ is the corresponding component of the bulk strain-rate tensor. While the denominator in Equation (37) can be replaced by Glen's isotropic flow law, the numerator requires an anisotropic rheology that depends on the COF state. The latter can be estimated by averaging a transversely isotropic monocrystal rheology over all grain orientations, with monocrystal rheological parameters chosen such that observed, bulk

directional enhancements are reproduced [37, 38]. For this purpose, a linear-viscous monocrystal rheology suffices upon carefully selecting a polycrystal stress–strain-rate homogenisation scheme in which the strain-rate tensor effectively depends on the second- and fourth-order structure tensors $\langle \mathbf{c}^2 \rangle$ and $\langle \mathbf{c}^4 \rangle$, defined as the average over the second and fourth outer product of the individual crystal orientations [38].

Notice that due to division in Equation (37), the enhancement factors are independent of the isotropic rate factor A and the magnitude of of the stress tensor. The same applies, in principle, to other isotropic contributions to the viscosity, such as temperature and impurities, insofar so they can be captured by modifying A. Finally, we note that in the case of a single-maximum or girdle COF, the principal COF directions can be taken without ambiguity to be the eigenvectors of $\mathbf{a}^{(2)}$. If one eigenvector is aligned with the vertical z-direction, $\mathbf{a}^{(2)}$ can be written in its eigenbasis as

$$\mathbf{a}^{(2)} = \begin{pmatrix} \lambda_\mathrm{x} & 0 & 0 \\ 0 & \lambda_\mathrm{y} & 0 \\ 0 & 0 & \lambda_\mathrm{z} \end{pmatrix}. \tag{38}$$

3.1 Estimating the second-order structure tensor from horizontal anisotropy

Our observational methodologies allow for inferring the difference in horizontal eigenvalues only, $\Delta\lambda = \lambda_\mathrm{y} - \lambda_\mathrm{x}$. However, by applying a few assumptions about the COF type to be expected over the different regions of NEGIS, guided by ice-core COF observations from EGRIP and the NEGIS shear margin, $\mathbf{a}^{(2)}$ can be estimated from $\Delta\lambda$. We distinguish between three different flow regimes:

1. **Inside the ice stream**: both the COF evolution model and the EGRIP ice core indicate the presence of a girdle in the ice stream centre so that $\lambda_\mathrm{x} \approx 0$. If combined with the definitions $\lambda_\mathrm{x} + \lambda_\mathrm{y} + \lambda_\mathrm{z} = 1$ and $\Delta\lambda = \lambda_\mathrm{y} - \lambda_\mathrm{x}$, it follows that:

$$\begin{aligned} \lambda_\mathrm{x} &= 0, \\ \lambda_\mathrm{y} &= \Delta\lambda, \\ \lambda_\mathrm{z} &= 1 - \Delta\lambda, \end{aligned} \tag{39}$$

or equivalently

$$\mathbf{a}^{(2)} = \begin{pmatrix} 0 & 0 & 0 \\ 0 & \Delta\lambda & 0 \\ 0 & 0 & 1-\Delta\lambda \end{pmatrix}. \tag{40}$$

2. **In the vicinity of the shear margins:** Ice cores and our COF evolution modelling results indicate the formation of strong horizontal single-maximum COFs in the shear margin and its vicinity, with the largest-eigenvalue eigenvector pointing approximately perpendicular to the shear margin. We thus propose that $\lambda_\mathrm{x} \approx \lambda_\mathrm{z}$ in a 4 km wide shear zone, from which it follows that

$$\begin{aligned} \lambda_\mathrm{x} \approx \lambda_\mathrm{z} &\approx \tfrac{1-\Delta\lambda}{3}, \\ \lambda_\mathrm{y} &\approx \tfrac{1+2\Delta\lambda}{3}, \end{aligned} \tag{41}$$

and therefore

$$\mathbf{a}^{(2)} = \begin{pmatrix} \frac{1-\Delta\lambda}{3} & 0 & 0 \\ 0 & \frac{1+2\Delta\lambda}{3} & 0 \\ 0 & 0 & \frac{1-\Delta\lambda}{3} \end{pmatrix}. \tag{42}$$

3. **Outside the shear margins:** In this regime, we lack direct observations from ice cores. Modelling results suggest that λ_x is small, but not zero. We therefore assume the more relaxed

condition $\lambda_x \approx 0.1$, from which it follows that

$$\begin{aligned}\lambda_x &= 0.1,\\ \lambda_y &= 0.1 + \Delta\lambda,\\ \lambda_z &= 0.8 - \Delta\lambda,\end{aligned} \tag{43}$$

and therefore

$$\mathbf{a}^{(2)} = \begin{pmatrix} 0.1 & 0 & 0 \\ 0 & 0.1 + \Delta\lambda & 0 \\ 0 & 0 & 0.8 - \Delta\lambda \end{pmatrix}. \tag{44}$$

3.2 Estimating the fourth-order structure tensor from the second-order tensor

In order to estimate the directional enhancement factors, both $\mathbf{a}^{(2)}$ and $\mathbf{a}^{(4)}$ must be known. Although our methodology is restricted in only deriving $\mathbf{a}^{(2)}$ from radar observations, $\mathbf{a}^{(4)}$ can be calculated from correlations with $\mathbf{a}^{(2)}$, derived from ice-core COFs and COF modelling, as elaborated on in the following.

The second- and fourth-order structure tensors can be calculated from the second- ($l = 2$) and fourth-order ($l = 4$) complex expansion coefficients of the orientation distribution function (ODF) [37]:

$$\mathrm{ODF}(\vartheta, \varphi) = \frac{1}{N} \sum_{l=0}^{\infty} \sum_{m=-l}^{l} \psi_l^m Y_l^m(\vartheta, \varphi), \tag{45}$$

where Y_l^m are the spherical harmonic expansion functions, depending on the co-latitude ϑ and longitude φ, and ψ_l^m are the complex expansion coefficients. In the case of a perfect single-maximum or girdle COF (as considered here), the independent expansion coefficient reduces to just two real numbers. This follows immediately from the fact that if the COF is rotated into a frame where it is horizontally symmetric, only ψ_2^0 and ψ_4^0 can be nonzero (only the modes Y_l^0 are rotationally symmetric around the z axis). As a consequence, if a correlation can be constructed between ψ_2^0 and ψ_4^0, then $\mathbf{a}^{(4)}$ can be constructed from $\mathbf{a}^{(2)}$ for single maximum and girdle COFs since $\mathbf{a}^{(2)}$ depends exclusively on ψ_2^m [37].

Figure 14 shows a scatter plot of measured correlations (markers) between ψ_2^0 and ψ_4^0, calculated from published c-axis data from ice cores drilled at domes and extensional/flank flows sites (references provided in the figure text). Note that all c-axes distributions have been rotated into a frame where they are approximately rotationally symmetric around the z-axis for this method to work; of course, once the correlation has been applied to determine ψ_4^0, the resulting ODF can be rotated back into its original orientation.

The brown and petrol solid lines in Fig. 14 show the corresponding modelled effect of lattice rotation (strain-induced rotation of c-axes) using the model by Rathmann *et al.* [37] for unconfined extension (brown) leading to a perfect girdle, and for unconfined compression (petrol) leading to a perfect single maximum. Although the observed correlation for single-maximum COFs falls slightly below the model line, COF samples from the EGRIP ice core (purple triangles) agree well with the modelled correlation, as does the NEGIS shear margin ice core (purple squares). In this work, we, therefore, assume that the correlation between ψ_2^0 and ψ_4^0 is given exactly by the modelled correlation (brown and petrol solid lines). We find, however, that the effect of discontinuous migration recrystallisation (DDRX; red solid line) and continuous recrystallisation (CDRX; black solid line) might explain the discrepancy between observed and modelled correlations elsewhere. Figs. 15–17 show the resulting enhancement factors in the eigenframe for radar-derived results and comparison between the eigenframe and flowframe for the Elmer/Ice results.

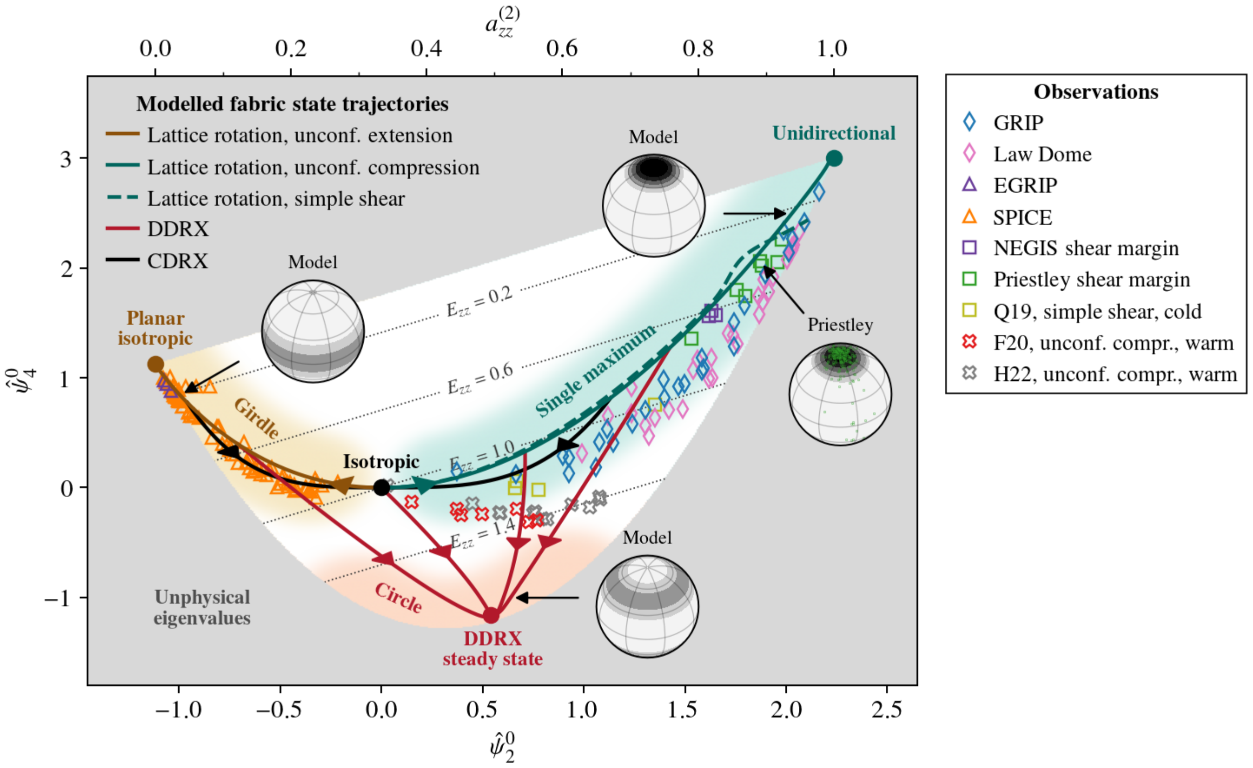

Figure 14: **Correlation between second- and fourth-order spectral coefficients.** A correlation between the fourth-order spectral coefficient, ψ_4^0, and the second-order coefficient, ψ_2^0, can be established for crystal orientation fabrics (COF) with horizontal isotropy. Ice-core girdle COFs from EGRIP (East Greenland ice-core project) [39] and SPICE (South Pole Ice Core Project) [41] follow the model-predicted correlation for unconfined extension (solid brown line). For single maximum COFs, ice-core data [42–44] and deformation tests [45, 46] tend to fall slightly below the modelled correlation line predicted for unconfined compression (solid petrol line) and simple shear (dashed petrol line), possibly due to the influence of discontinuous dynamic recrystallisation (DDRX) or rotation recrystallisation (CDRX) (modelled effects indicated by red and black solid lines, respectively; see main text). Both cores taken in our survey area (purple markers), however, agree well with the modelled correlation. Grey dashed contours show modelled enhancement factors for vertical compression/extension, E_{zz}. Grey regions indicate nonphysical eigenvalues for horizontally isotropic orientation distribution functions (ODF).

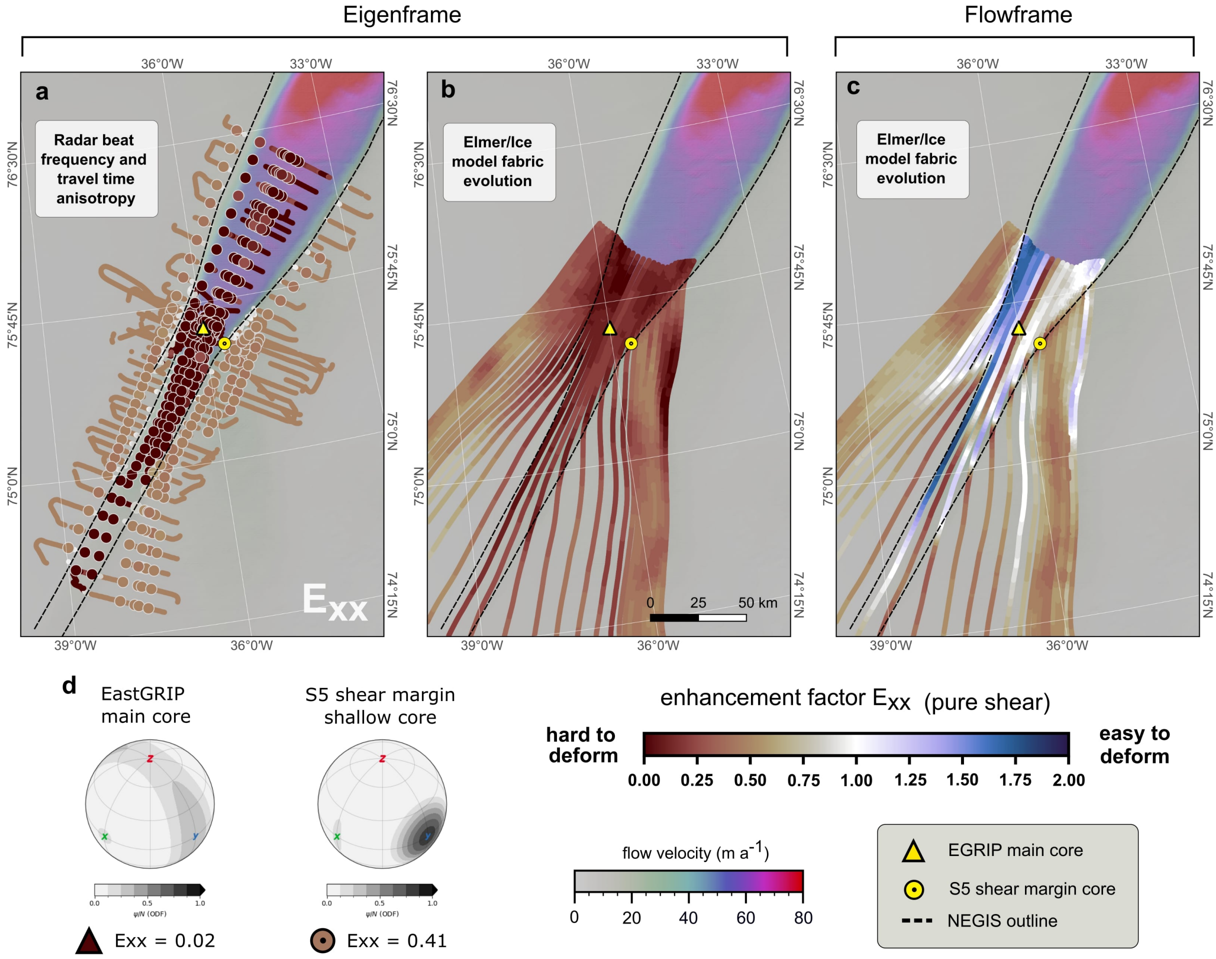

Figure 15: **Enhancement factor E_{xx} (pure shear along ice flow).** Panel **a** shows the enhancement factors derived from the radar measurements from the crosspoint analysis (larger dots with white rim) and beat-signature analysis (small dots without rim). Panels **b** and **c** show the enhancement factors derived from the Elmer/Ice crystal orientation fabric (COF) evolution modelling. Note that Panels **a** and **b** represent the enhancement factors with respect to the eigenframe and panel **c** with respect to the flowframe. The stereographic projections of the c-axes and enhancement factors (in eigenframe) derived from the two ice-core locations (East Greenland ice-core project (EGRIP) main core and S5 shear margin core) are shown in **d**. Background shows satellite-based surface velocities [29].

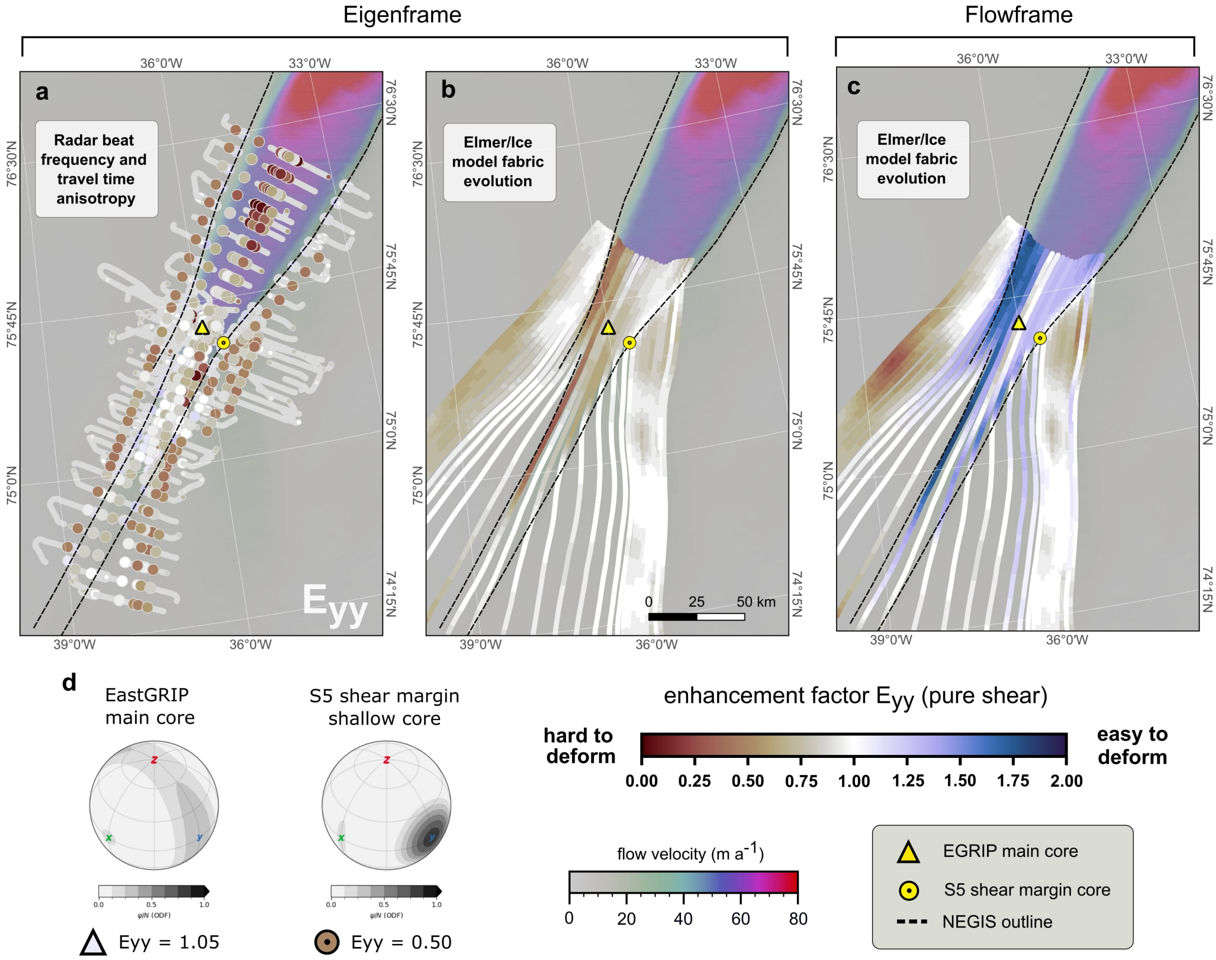

Figure 16: **Enhancement factor E_{yy} (pure shear perpendicular to ice flow).** Panel **a** shows the enhancement factors derived from the radar measurements from the crosspoint analysis (larger dots with white rim) and beat-signature analysis (smaller dots without rim). Panels **b** and **c** show the enhancement factors derived from the Elmer/Ice crystal orientation fabric (COF) evolution modelling. Note that Panels **a** and **b** represent the enhancement factors with respect to the eigenframe and panel **c** with respect to the flowframe. The respective stereographic projections of the c-axes and enhancement factors (in eigenframe) derived from the two ice-core locations (East Greenland ice-core project (EGRIP) main core and S5 shear margin core) are shown in **d**. Background shows satellite-based surface velocities [29].

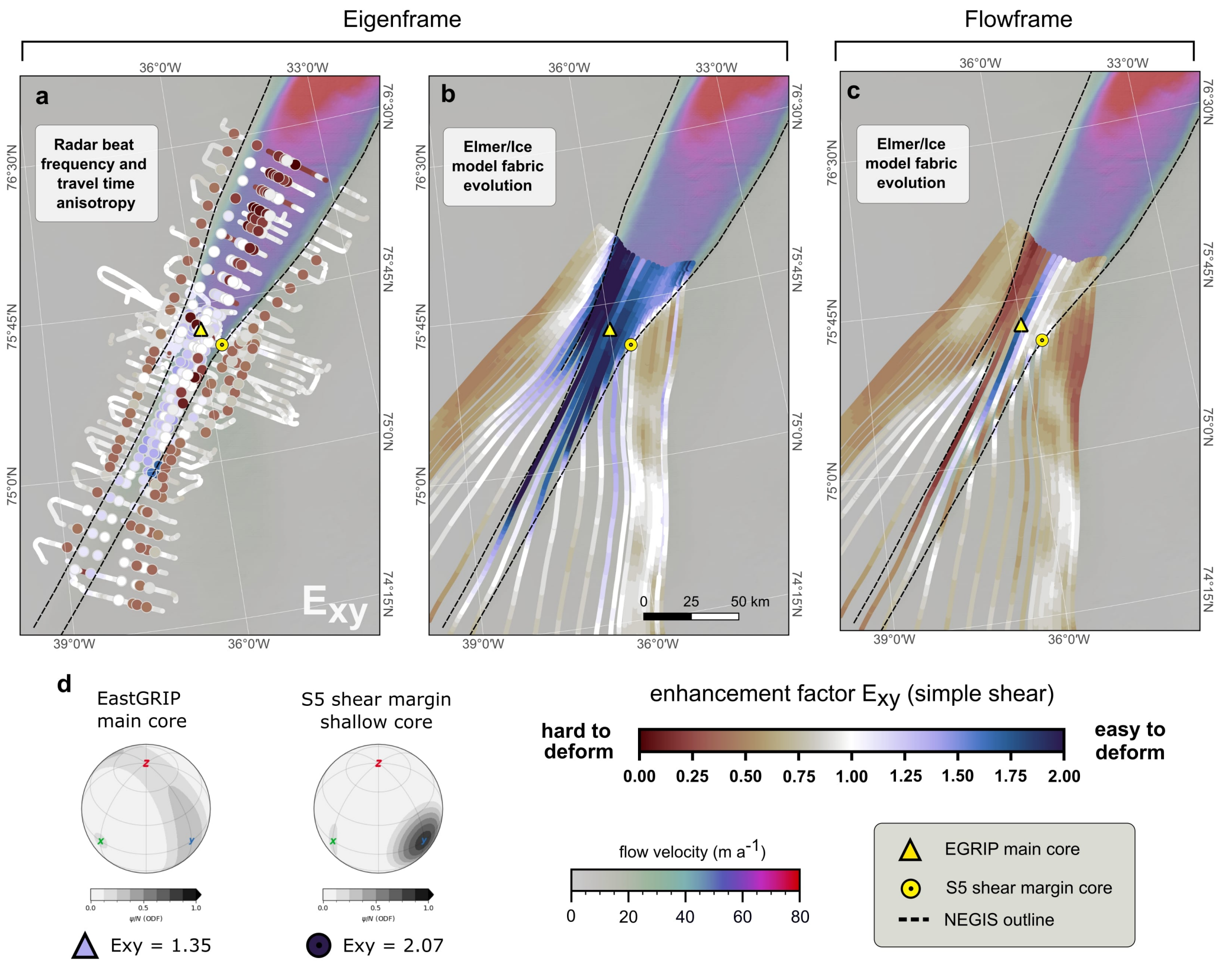

Figure 17: **Enhancement factor E_{xy} (simple shear along ice flow).** Panel **a** shows the enhancement factors derived from the radar measurements from the crosspoint analysis (larger dots with white rim) and beat frequency anisotropy analysis (smaller dots without rim). Panels **b** and **c** show the enhancement factors derived from the Elmer/Ice crystal orientation fabric (COF) evolution modelling. Note that Panels **a** and **b** represent the enhancement factors with respect to the eigenframe and panel **c** with respect to the flowframe. The respective stereographic projections of the c-axes and enhancement factors (in eigenframe) derived from the two ice-core locations (East Greenland ice-core project (EGRIP) main core and S5 shear margin core) are shown in **d**. Background shows satellite-based surface velocities [29].

4 Equivalent enhancement due to temperature

Temperature is, besides the COF, the most important factor controlling the ice viscosity. To evaluate the potential significance of either effect, we estimated the equivalent temperature difference required to obtain a similar enhancement in the corresponding direction from the enhancement factors provided above. Modified to the effect of temperature, Equation (37) becomes:

$$E = \frac{\dot{\epsilon}(\boldsymbol{\tau}, T_2)}{\dot{\epsilon}(\boldsymbol{\tau}, T_1)} = \frac{A(T_2)\boldsymbol{\tau}^n}{A(T_1)\boldsymbol{\tau}^n}. \tag{46}$$

At temperatures below –10 °C, the temperature dependence of the creep parameter A can be described by a simple Arrhenius relationship

$$E = \frac{A_0 \exp(-\frac{Q}{RT_2})\boldsymbol{\tau}^n}{A_0 \exp(-\frac{Q}{RT_1})\boldsymbol{\tau}^n} = \exp\left(\frac{Q}{R}\left(\frac{1}{T_1} - \frac{1}{T_2}\right)\right), \tag{47}$$

whereby the prefactor A_0 and the stress tensor $\boldsymbol{\tau}^n$ cancel out and R is the universal gas constant. Field observations and laboratory measurements show that the activation energy Q is approximately 60 kJmol^{-1} for temperatures below –10 °C [47].

The temperatures, T_1 and T_2 are given in Kelvin, and can be expressed as $\Delta T = T_2 - T_1$. Equation (47) can thus be re-written as

$$\Delta T = \frac{{T_1}^2 \frac{R}{Q} \ln(E)}{1 - T_1 \frac{R}{Q} \ln(E)}. \tag{48}$$

Borehole measurements in Greenland deep drilling sites show ice at cold conditions for the major part of the ice column [48]. Here, we assumed the reference temperature T_1 equals –20 °C (as also suggested by Elmer/Ice model results), although the exact choice of T_1 only has a minor effect on ΔT as long as it is in the cold-temperature range (below -10 °C) as shown in Fig. 18.

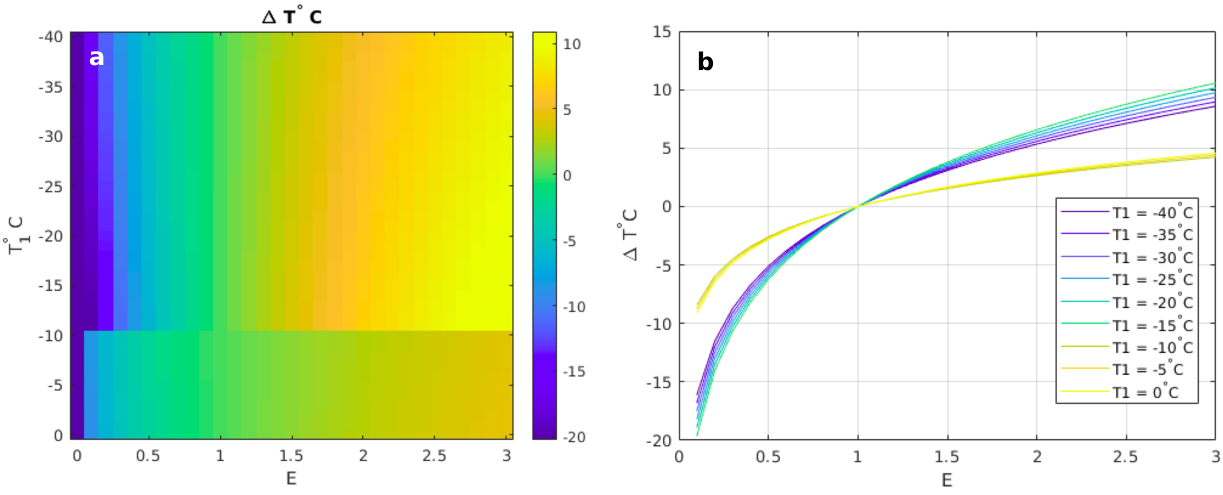

Figure 18: **Sensitivity of ΔT towards choice of reference temperature T_1.** **a** Temperature difference ΔT in degree Celcius for varying enhancement factors E (x-axis) and a given reference temperature T_1 (y-axis). **b** Sensitivity of ΔT on the choice of the reference temperature T_1. For reference temperatures, T_1 below –10 °C the activation energy Q is assumed to be 60 kJ mol^{-1} whereas for temperatures above –10 °C Q = 152 kJ mol^{-1} is assumed, leading to a stronger effect on the flow enhancement for smaller temperature variations than for colder ice. Within the two temperature regimes, the sensitivity to T_1 is minor.

In analogy to Fig. 15–17, the temperature differences, ΔT, that would be required to obtain equivalent enhancements as the COF for the corresponding deformation are shown in Fig. 19–21. For example, the COF causes along-flow stiffening for pure-shear deformation along flow which would correspond to ice inside the ice stream being 15–30 °C colder than the reference temperature

(Fig. 19a) if a similar low viscosity should be explained by temperature only (assuming isotropic ice). In the shear margins, COF-induced softening by a factor of two for horizontal shear deformation could also be achieved by shear heating of 5 °C (Fig. 21b). In other words, the COF has the potential to change the effective viscosities by orders of magnitude which can not realistically be explained by temperature effects in some areas, particularly the along-flow stiffening inside the ice stream. Temperature could, however, play an equally important role in softening the shear margins as the COF since a similar enhancement factor could be obtained by realistic temperature anomalies expected by shear-heating. For warm-ice conditions (above -10 °C) which could be present near the bed or in shear zones, however, the assumed value of the activation energy Q no longer holds, implying that similar enhancements could be achieved by smaller temperature anomalies.

5 Characteristic time

The characteristic time for a viscous response is defined as

$$\tau_{\mathrm{char}} = \frac{(2+2\mu)\eta}{Y}, \tag{49}$$

where the Poisson ratio μ in ice can be assumed to be 0.325, and the Young's modulus Y is approximately 10^9 Pa. An enhancement factor of 0.1, equivalent to the viscosity η being ten times larger than for isotropic ice, would imply that the characteristic time is ten times larger. Therefore, a viscous deformation happens ten times slower in ice which is harder for pure-shear deformation along flow in comparison to isotropy. A change in basal stress, for example, due to changes in water pressure below the ice, is always causing an instantaneous elastic response, but with harder ice, the viscous response is further delayed.

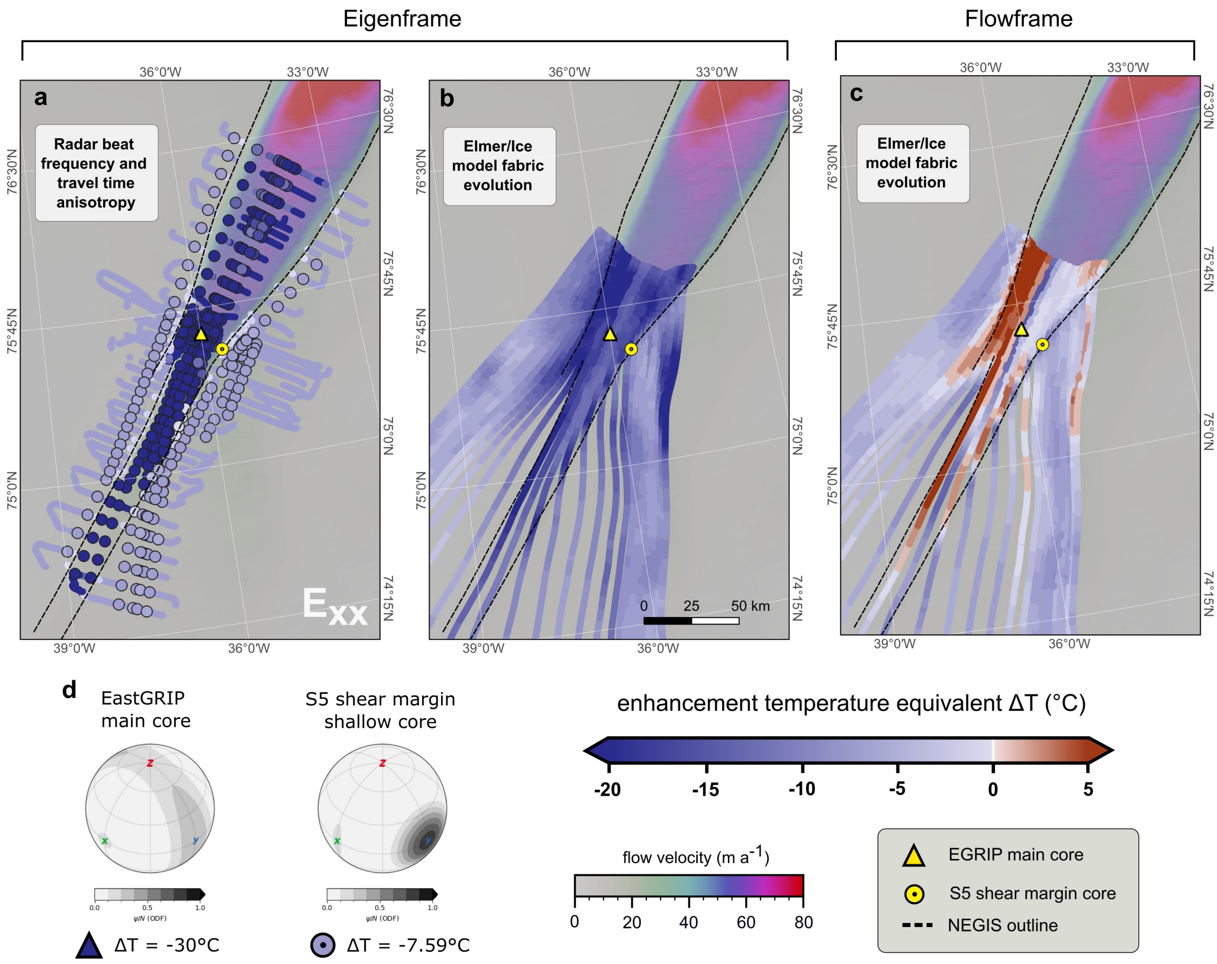

Figure 19: **E_{xx}-equivalent temperature difference ΔT.** Temperature anomaly ΔT which would be required to obtain an (isotropic) flow enhancement as suggested by the crystal orientation fabric (COF) for pure-shear deformation along flow (E_{xx}). Akin to Fig. 15, panel **a** shows the temperature anomalies equivalent to the enhancements obtained from radar observations (larger dots with black rim corresponding to crosspoint analysis, smaller dots without rim corresponding to beat-signature analysis), **b** and **c** those for the modelling results in eigenframe and flowframe respectively, and **d** corresponds to the observations in the two ice cores. Temperature anomalies are calculated for a reference temperature of –20 °C. Background shows satellite-based surface velocities [29].

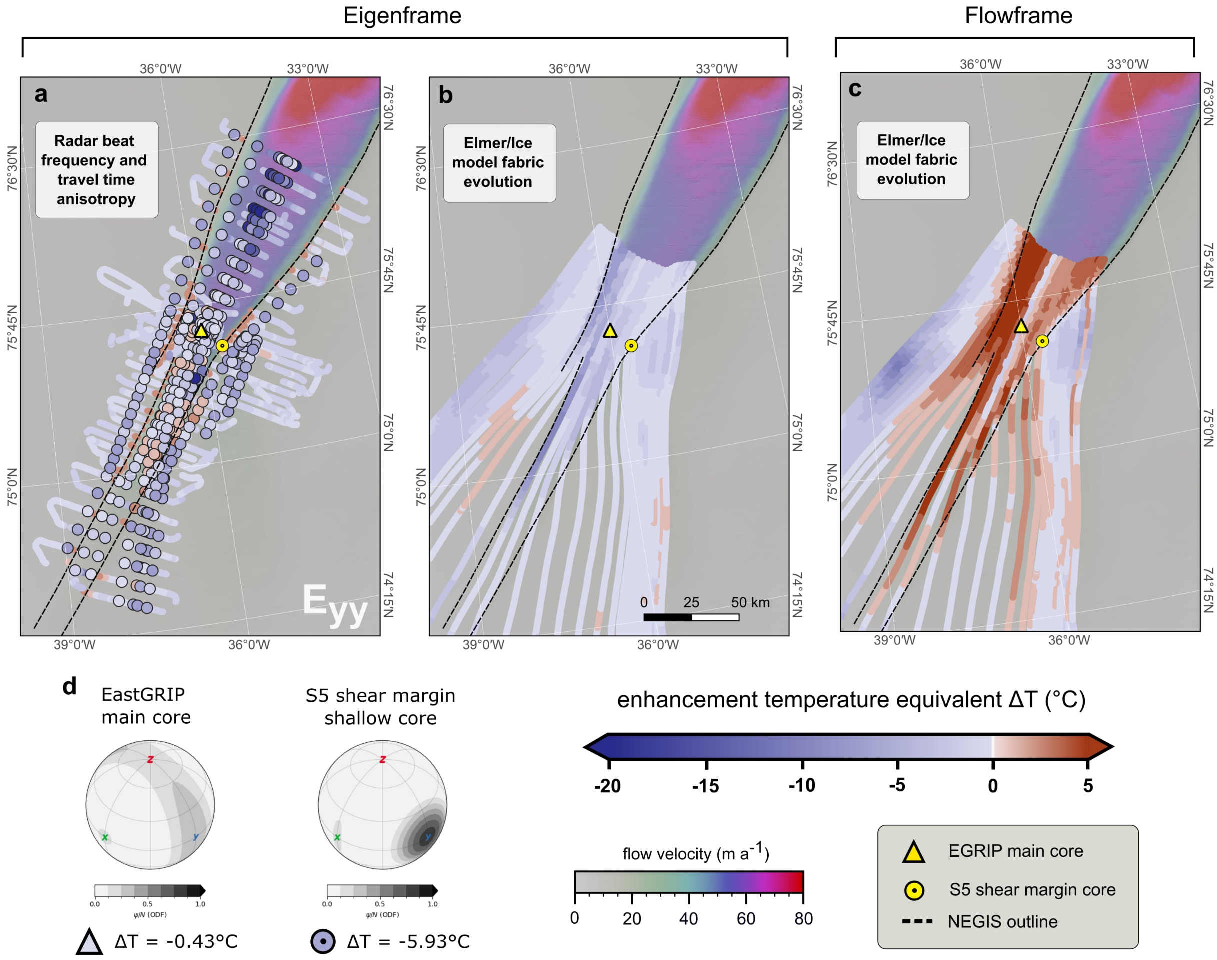

Figure 20: **E_{yy}-equivalent temperature difference ΔT.** Temperature anomaly ΔT which would be required to obtain an (isotropic) flow enhancement as suggested by the crystal orientation fabric (COF) for pure-shear deformation perpendicular to flow (E_{yy}). Akin to Fig. 16, panel **a** shows the temperature anomalies equivalent to the enhancements obtained from radar observations (larger dots with black rim corresponding to crosspoint analysis, smaller dots without rim corresponding to beat-frequency analysis), **b** and **c** those for the modelling results in eigenframe and flowframe respectively, and **d** corresponds to the observations in the two ice cores. Temperature anomalies are calculated for a reference temperature of –20 °C. Background shows satellite-based surface velocities [29].

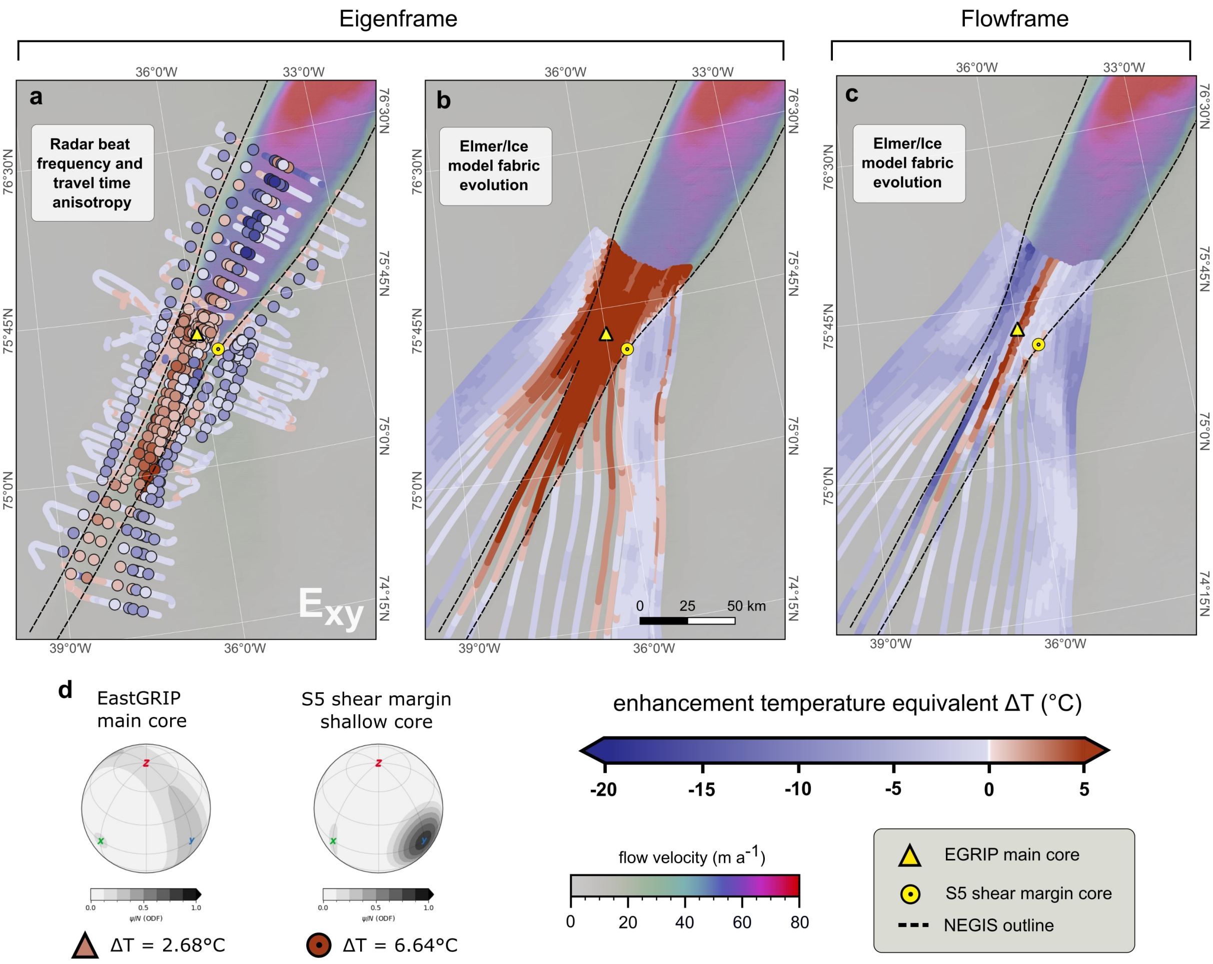

Figure 21: **E_{xy}-equivalent temperature difference ΔT.** Temperature anomaly ΔT which would be required to obtain an (isotropic) flow enhancement as suggested by the crystal orientation fabric (COF) for horizontal simple-shear deformation (E_{xy}). Akin to Fig. 17, panel **a** shows the temperature anomalies equivalent to the enhancements obtained from radar observations (larger dots with black rim corresponding to crosspoint analysis, smaller dots without rim corresponding to beat-signature analysis), **b** and **c** those for the modelling results in eigenframe and flowframe respectively, and **d** corresponds to the observations in the two ice cores. Temperature anomalies are calculated for a reference temperature of –20 °C. Background shows satellite-based surface velocities [29].

6 Notation (not exhaustive)

x', y'	geographic east, north
$\tilde{\mathrm{x}}, \tilde{\mathrm{y}}$	direction parallel, transverse to ice flow
$\hat{\mathrm{x}}, \hat{\mathrm{y}}$	flow-parallel, flow perpendicular radar antenna polarisation
x, y	direction of smaller, larger horizontal eigenvalue
z	depth below ice sheet surface
θ_{flow}	angle between ice flow direction and largest horizontal COF eigenvector
θ_{geo}	angle between geographic east and largest horizontal COF eigenvector
θ_{pol}	angle between radar polarisations and horizontal COF eigenvectors
β	angle between geographic east and ice flow direction
δ	angle between two radar polarisation directions
$\mathbf{a}^{(2)}, \mathbf{a}^{(4)}$	second, fourth-order COF orientation tensor
$\mathbf{a}_{1,2,3}$	COF eigenvectors
$\lambda_{1,2,3}, \lambda_{\mathrm{x,y,z}}$	COF eigenvalues of increasing size, in x, y, z direction
$\varepsilon*$	complex dielectric constant
$\varepsilon, \varepsilon_0$	relative dielectric permittivity, permittivity of free space
γ	electrical conductivity
κ	thermal conductivity
ω	angular frequency
$\boldsymbol{\varepsilon}^{\mathrm{m}}, \boldsymbol{\varepsilon}$	relative dielectric permittivity tensor for monocrystal, polycrystal
$\varepsilon^{\mathrm{m}}_{\parallel}, \varepsilon^{\mathrm{m}}_{\perp}$	dielectric permittivity parallel, perpendicular to the c-axis
$\Delta\varepsilon^{\mathrm{m}}, \Delta\varepsilon$	relative dielectric anisotropy of a monocrystal, polycrystal
$\varepsilon_{\mathrm{xy}}$	horizontal mean of relative dielectric permittivity of a polycrystal
$\Delta\varepsilon_{\mathrm{a}}$	apparent horizontal dielectric anisotropy between radar polarisations $\tilde{\mathrm{x}}$ and $\tilde{\mathrm{y}}$
$\Delta\lambda_{\mathrm{a}}$	apparent difference in horizontal eigenvalues between radar polarisation directions
$\Delta\lambda$	difference in horizontal eigenvalues
c, c_0	electromagnetic wave speed, speed of light
$e_{\mathrm{x}}, e_{\mathrm{y}}$	electromagnetic wave components in principal COF directions x and y
$t_{\hat{\mathrm{x}}}, t_{\hat{\mathrm{y}}}$	two-way travel time for $\hat{\mathrm{x}}$, $\hat{\mathrm{y}}$ polarised wave reflection
$\bar{t}, \Delta t$	mean, difference of two-way travel time
$\overline{\varepsilon_{\hat{\mathrm{x}}}}, \overline{\varepsilon_{\hat{\mathrm{y}}}}$	depth-averaged directional permittivities
ϕ	phase difference between ordinary and extraordinary wave
f	centre frequency of radar system
k	wave number
$f_{\mathrm{mod}}, T_{\mathrm{mod}}, k_{\mathrm{mod}}$	power modulation frequency, period, wave number
s	propagated path of electromagnetic wave
$\theta_{\mathrm{mod}}, l_{\mathrm{mod}}$	phase, wavelength of beat signature
$u_{\mathrm{x}}, u_{\mathrm{y}}, u_{\mathrm{z}}$	ice flow velocities flow-parallel, flow-transverse, vertical
$\boldsymbol{\sigma}$	Cauchy stress tensor

$\rho_{\rm i}$	density of ice
g	force of gravity
$\boldsymbol{\tau}$	deviatoric stress tensor
$\mathbf{M}_{1,2,3}$	structure tensors
n	flow law exponent
A	flow-law prefactor
T	temperature
$W(\tilde{x})$	width of flow band
$\mathbf{W}$	spin tensor
$\dot{\boldsymbol{\epsilon}}$	strain rate tensor
Ψ	strain heating
r	flow line curvature
H	ice thickness
η	ice viscosity
q, κ	heat capacity, thermal conductivity of ice
$E_{\rm vw}$, E	flow enhancement factor in direction *vw*, isotropic enhancement factor
$E'_{\rm cc}$, $E'_{\rm ca}$	enhancement of a monocrystal along the c-axis, basal plane
$\mathbf{c}_i$	c-axis orientation of monocrystal
ψ_l^m	complex spectral expansion coefficient
Y_l^m	spherical harmonics expansion function
R	universal gas constant
Q	activation energy
$\tau_{\rm char}$	characteristic time
μ, Y	Poisson ratio, Young's modulus

Supplementary References

1. Matsuoka, T., Fujita, S., Morishima, S. & Mae, S. Precise measurement of dielectric anisotropy in ice Ih at 39 GHz. *Journal of Applied Physics* **81,** 2344–2348 (1997).
2. Fujita, S., Maeno, H. & Matsuoka, K. Radio-wave depolarization and scattering within ice sheets: a matrix-based model to link radar and ice-core measurements and its application. *Journal of Glaciology* **52,** 407–424 (2006).
3. Franke, S., Jansen, D., Binder, T., Paden, J. D., Dörr, N., Gerber, T. A., Miller, H., Dahl-Jensen, D., Helm, V., Steinhage, D., Weikusat, I., Wilhelms, F. & Eisen, O. Airborne ultra-wideband radar sounding over the shear margins and along flow lines at the onset region of the Northeast Greenland Ice Stream. *Earth System Science Data* **14,** 763–779 (2022).
4. Mojtabavi, S., Wilhelms, F., Cook, E., Davies, S. M., Sinnl, G., Skov Jensen, M., Dahl-Jensen, D., Svensson, A., Vinther, B. M., Kipfstuhl, S., Jones, G., Karlsson, N. B., Faria, S. H., Gkinis, V., Kjær, H. A., Erhardt, T., Berben, S. M. P., Nisancioglu, K. H., Koldtoft, I. & Rasmussen, S. O. A first chronology for the East Greenland Ice-core Project (EGRIP) over the Holocene and last glacial termination. *Climate of the Past* **16,** 2359–2380 (2020).
5. Wilhelms, F., Kipfstuhl, J., Miller, H., Heinloth, K. & Firestone, J. Precise dielectric profiling of ice cores: a new device with improved guarding and its theory. *Journal of Glaciology* **44,** 171–174 (1998).
6. Mojtabavi, S., Eisen, O., Franke, S., Jansen, D., Steinhage, D., Paden, J., Dahl-Jensen, D., Weikusat, I., Eichler, J. & Wilhelms, F. Origin of englacial stratigraphy at three deep ice core sites of the Greenland Ice Sheet by synthetic radar modelling. *Journal of Glaciology,* 1–13 (2022).
7. Riverman, K. L., Alley, R. B., Anandakrishnan, S., Christianson, K., Holschuh, N. D., Medley, B., Muto, A. & Peters, L. E. Enhanced Firn Densification in High-Accumulation Shear Margins of the NE Greenland Ice Stream. *Journal of Geophysical Research: Earth Surface* **124,** 365–382 (2019).
8. Vallelonga, P., Christianson, K., Alley, R. B., Anandakrishnan, S., Christian, J. E. M., Dahl-Jensen, D., Gkinis, V., Holme, C., Jacobel, R. W., Karlsson, N. B., Keisling, B. A., Kipfstuhl, S., Kjær, H. A., Kristensen, M. E. L., Muto, A., Peters, L. E., Popp, T., Riverman, K. L., Svensson, A. M., Tibuleac, C., Vinther, B. M., Weng, Y. & Winstrup, M. Initial results from geophysical surveys and shallow coring of the Northeast Greenland Ice Stream (NEGIS). *The Cryosphere* **8,** 1275–1287 (2014).
9. Christianson, K., Peters, L. E., Alley, R. B., Anandakrishnan, S., Jacobel, R. W., Riverman, K. L., Muto, A. & Keisling, B. A. Dilatant till facilitates ice-stream flow in northeast Greenland. *Earth and Planetary Science Letters* **401,** 57–69 (2014).
10. Oraschewski, F. M. & Grinsted, A. Modeling enhanced firn densification due to strain softening. *The Cryosphere* **16,** 2683–2700 (2022).
11. Young, T., Schroeder, D. M., Jordan, T. M., Christoffersen, P., Tulaczyk, S. M., Culberg, R. & Bienert, N. L. Inferring ice fabric from birefringence loss in airborne radargrams: Application to the eastern shear margin of Thwaites Glacier, West Antarctica. *Journal of Geophysical Research: Earth Surface* **126,** e2020JF006023 (2021).
12. Ershadi, M. R., Drews, R., Martıín, C., Eisen, O., Ritz, C., Corr, H., Christmann, J., Zeising, O., Humbert, A. & Mulvaney, R. Polarimetric radar reveals the spatial distribution of ice fabric at domes and divides in East Antarctica. *The Cryosphere* **16,** 1719–1739 (2022).
13. Jordan, T. M., Martín, C., Brisbourne, A. M., Schroeder, D. M. & Smith, A. M. Radar characterization of ice crystal orientation fabric and anisotropic viscosity within an Antarctic ice stream. *Journal of Geophysical Research: Earth Surface* **127,** e2022JF006673 (2022).
14. Jordan, T. M., Schroeder, D. M., Castelletti, D., Li, J. & Dall, J. A polarimetric coherence method to determine ice crystal orientation fabric from radar sounding: application to the NEEM Ice Core Region. *IEEE Transactions on Geoscience and Remote Sensing* **57,** 8641–8657 (2019).

15. Yan, J.-B., Li, L., Nunn, J. A., Dahl-Jensen, D., O'Neill, C., Taylor, R. A., Simpson, C. D., Wattal, S., Steinhage, D., Gogineni, P., Miller, H. & Eisen, O. Multiangle, Frequency, and Polarization Radar Measurement of Ice Sheets. *IEEE Journal of Selected Topics in Applied Earth Observations and Remote Sensing* **13,** 2070–2080 (2020).
16. Gardner, A., Fahnestock, M. & Scambos, T. ITS_LIVE regional glacier and ice sheet surface velocities. *Data archived at National Snow and Ice Data Center.* https://doi.org/10.5067/6II6VW8LLWJ7 (2021).
17. Zeising, O., Gerber, T. A., Eisen, O., Ershadi, M. R., Stoll, N., Weikusat, I. & Humbert, A. Improved estimation of the bulk ice crystal fabric asymmetry from polarimetric phase co-registration. *The Cryosphere* **17,** 1097–1105 (2023).
18. Stewart, C. L., Christoffersen, P., Nicholls, K. W., Williams, M. J. & Dowdeswell, J. A. Basal melting of Ross Ice Shelf from solar heat absorption in an ice-front polynya. *Nature Geoscience* **12,** 435 (2019).
19. Vaňková, I., Nicholls, K. W., Corr, H. F., Makinson, K. & Brennan, P. V. Observations of tidal melt and vertical strain at the Filchner-Ronne Ice Shelf, Antarctica. *Journal of Geophysical Research: Earth Surface* **125,** e2019JF005280 (2020).
20. Zeising, O. & Humbert, A. Indication of high basal melting at the EastGRIP drill site on the Northeast Greenland Ice Stream. *The Cryosphere* **15,** 3119–3128 (2021).
21. Stewart, C. L. *Ice-ocean interactions beneath the north-western Ross Ice Shelf, Antarctica* PhD thesis (University of Cambridge, 2018).
22. Gillet-Chaulet, F., Gagliardini, O., Meyssonnier, J., Montagnat, M. & Castelnau, O. A user-friendly anisotropic flow law for ice-sheet modelling. *Journal of Glaciology* **51,** 3–14 (2005).
23. Ma, Y., Gagliardini, O., Ritz, C., Gillet-Chaulet, F., Durand, G. & Montagnat, M. Enhancement factors for grounded ice and ice shelves inferred from an anisotropic ice-flow model. *Journal of Glaciology* **56,** 805–812 (2010).
24. Castelnau, O., Shoji, H., Mangeney, A., Milsch, H., Duval, P., Miyamoto, A., Kawada, K. & Watanabe, O. Anisotropic behavior of GRIP ices and flow in Central Greenland. *Earth and Planetary Science Letters* **154,** 307–322 (1998).
25. Hvidberg, C. S. Steady-state thermomechanical modelling of ice flow near the centre of large ice sheets with the finite-element technique. *Annals of Glaciology* **23,** 116–123 (1996).
26. Gagliardini, O. & Meyssonnier, J. Analytical derivations for the behavior and fabric evolution of a linear orthotropic ice polycrystal. *Journal of Geophysical Research: Solid Earth* **104,** 17797–17809 (1999).
27. Gillet-Chaulet, F., Gagliardini, O., Meyssonnier, J., Zwinger, T. & Ruokolainen, J. Flow-induced anisotropy in polar ice and related ice-sheet flow modelling. *J. Non-Newtonian Fluid Mech* **134,** 33–43 (2006).
28. Reeh, N. A Flow-line Model for Calculating the Surface Profile and the Velocity, Strain-rate, and Stress Fields in an Ice Sheet. *Journal of Glaciology* **34,** 46–55 (1988).
29. Joughin, I., Smith, B. E. & Howat, I. M. A complete map of Greenland ice velocity derived from satellite data collected over 20 years. *Journal of Glaciology* **64,** 1–11 (2018).
30. Joughin, I. R., Smith, B. E., Howat, I. M., Moon, T. & Scambos, T. A. A SAR record of early 21st century change in Greenland. *Journal of Glaciology* **62,** 1–10 (2016).
31. Noël, B., Van De Berg, W. J., Van Meijgaard, E., Kuipers Munneke, P., Van De Wal, R. S. W. & Van Den Broeke, M. R. Evaluation of the updated regional climate model RACMO2.3 : Summer snowfall impact on the Greenland Ice Sheet. *The Cryosphere* **9,** 1831–1844 (2015).
32. MacGregor, J. A., Fahnestock, M. A., Catania, G. A., Aschwanden, A., Clow, G. D., Colgan, W. T., Gogineni, S. P., Morlighem, M., Nowicki, S. M. J., Paden, J. D., Price, S. F. & Seroussi, H. A synthesis of the basal thermal state of the Greenland Ice Sheet. *Journal of Geophysical Research: Earth Surface* **121,** 1328–1350 (2016).

33. Raymond, C. F. Deformation in the Vicinity of Ice Divides. *Journal of Glaciology* **29,** 357–373 (1983).

34. Holschuh, N., Lilien, D. A. & Christianson, K. Thermal Weakening, Convergent Flow, and Vertical Heat Transport in the Northeast Greenland Ice Stream Shear Margins. *Geophysical Research Letters* **46,** 8184–8193 (2019).

35. Wang, Y., Thorsteinsson, T., Kipfstuhl, J., Miller, H., Dahl-Jensen, D. & Shoji, H. A vertical girdle fabric in the NorthGRIP deep ice core, North Greenland. *Annals of Glaciology* **35,** 515–520 (2002).

36. Davies, J. H. Global map of solid Earth surface heat flow. *Geochemistry, Geophysics, Geosystems* **14,** 4608–4622 (2013).

37. Rathmann, N. M., Hvidberg, C. S., Grinsted, A., Lilien, D. A. & Dahl-Jensen, D. Effect of an orientation-dependent non-linear grain fluidity on bulk directional enhancement factors. *Journal of Glaciology* **67,** 569–575 (2021).

38. Rathmann, N. M. & Lilien, D. A. Inferred basal friction and mass flux affected by crystal-orientation fabrics. *Journal of Glaciology,* 1–17 (2021).

39. Westhoff, J., Stoll, N., Franke, S., Weikusat, I., Bons, P., Kerch, J., Jansen, D., Kipfstuhl, S. & Dahl-Jensen, D. A stratigraphy-based method for reconstructing ice core orientation. *Annals of Glaciology* **62,** 191–202 (2021).

40. Thorsteinsson, T. An analytical approach to deformation of anisotropic ice-crystal aggregates. *Journal of Glaciology* **47,** 507–516 (2001).

41. Voigt, D. c-axis fabric of the South pole ice core. *SPC14" U.S. Antarctic Program (USAP) Data Center.* https://doi.org/10.15784/601057 (2017).

42. Thomas, R. E., Negrini, M., Prior, D. J., Mulvaney, R., Still, H., Bowman, M. H., Craw, L., Fan, S., Hubbard, B., Hulbe, C., Kim, D. & Lutz, F. Microstructure and Crystallographic Preferred Orientations of an Azimuthally Oriented Ice Core from a Lateral Shear Margin: Priestley Glacier, Antarctica. *Frontiers in Earth Science* **9** (2021).

43. Thorsteinsson, T., Kipfstuhl, J. & Miller, H. Textures and fabrics in the GRIP ice core. *Journal of Geophysical Research: Oceans* **102,** 26583–26599 (1997).

44. Treverrow, A., Jun, L. & Jacka, T. H. Ice crystal *c*-axis orientation and mean grain size measurements from the Dome Summit South ice core, Law Dome, East Antarctica. *Earth System Science Data* **8,** 253–263 (2016).

45. Fan, S., Hager, T. F., Prior, D. J., Cross, A. J., Goldsby, D. L., Qi, C., Negrini, M. & Wheeler, J. Temperature and strain controls on ice deformation mechanisms: insights from the microstructures of samples deformed to progressively higher strains at -10, -20 and -30°C. *The Cryosphere* **14,** 3875–3905 (2020).

46. Qi, C., Prior, D. J., Craw, L., Fan, S., Llorens, M.-G., Griera, A., Negrini, M., Bons, P. D. & Goldsby, D. L. Crystallographic preferred orientations of ice deformed in direct-shear experiments at low temperatures. *The Cryosphere* **13,** 351–371 (2019).

47. Weertman, J. Creep deformation of ice. *Annual Review of Earth and Planetary Sciences* **11,** 215–240 (1983).

48. Dahl-Jensen, D., Mosegaard, K., Gundestrup, N., Clow, G. D., Johnsen, S. J., Hansen, A. W. & Balling, N. Past temperatures directly from the Greenland ice sheet. *Science* **282,** 268–271 (1998).
